# On the distribution of the number of internal equilibria in random evolutionary games

**DOI:** 10.1007/s00285-018-1276-0

**Published:** 2018-08-01

**Authors:** Manh Hong Duong, Hoang Minh Tran, The Anh Han

**Affiliations:** 1 School of Mathematics, University of Birmingham, Birmingham, B15 2TT UK; 2 Data Analytics Department, Esmart Systems, 1783 Halden, Norway; 3 School of Computing, Media & the Arts, Teesside University, Middlesbrough, TS1 3BX UK

**Keywords:** Evolutionary game theory, Multi-player games, Replicator dynamics, Random polynomials, Distributions of equilibria, Random games, 91A22, 91A15, 30C15

## Abstract

The analysis of equilibrium points is of great importance in evolutionary game theory with numerous practical ramifications in ecology, population genetics, social sciences, economics and computer science. In contrast to previous analytical approaches which primarily focus on computing the expected number of internal equilibria, in this paper we study the distribution of the number of internal equilibria in a multi-player two-strategy random evolutionary game. We derive for the first time a closed formula for the probability that the game has a certain number of internal equilibria, for both normal and uniform distributions of the game payoff entries. In addition, using Descartes’ rule of signs and combinatorial methods, we provide several universal upper and lower bound estimates for this probability, which are independent of the underlying payoff distribution. We also compare our analytical results with those obtained from extensive numerical simulations. Many results of this paper are applicable to a wider class of random polynomials that are not necessarily from evolutionary games.

## Introduction

### Motivation

Evolutionary Game Theory (EGT) (Maynard Smith and Price 1973) has become one of the most diverse and far reaching theories in biology finding its applications in a plethora of disciplines such as ecology, population genetics, social sciences, economics and computer science (Maynard Smith 1982; Axelrod 1984; Hofbauer and Sigmund 1998; Nowak 2006; Broom and Rychtář 2013; Perc and Szolnoki 2010; Sandholm 2010; Han et al. 2017), see also recent reviews (Wang et al. 2016; Perc et al. 2017). For example, in economics, EGT has been employed to make predictions in situations where traditional assumptions about agents’ rationality and knowledge may not be justified (Friedman 1998; Sandholm 2010). In computer science, EGT has been used extensively to model dynamics and emergent behaviour in multiagent systems (Helbing et al. 2015; Tuyls and Parsons 2007; Han 2013). Furthermore, EGT has provided explanations for the emergence and stability of cooperative behaviours which is one of the most well-studied and challenging interdisciplinary problems in science (Pennisi 2005; Hofbauer and Sigmund 1998; Nowak 2006). A particularly important subclass in EGT is random evolutionary games in which the payoff entries are random variables. They are useful to model social and biological systems in which very limited information is available, or where the environment changes so rapidly and frequently that one cannot describe the payoffs of their inhabitants’ interactions (May 2001; Fudenberg and Harris 1992; Han et al. 2012; Gross et al. 2009; Galla and Farmer 2013).

Similar to the foundational concept of Nash equilibrium in classical game theory (Nash 1950), the analysis of equilibrium points is of great importance in EGT. It provides essential understanding of complexity in a dynamical system, such as its behavioural, cultural or biological diversity (Haigh 1988, 1990; Broom et al. 1997; Broom 2003; Gokhale and Traulsen 2010, 2014; Han et al. 2012; Duong and Han 2015, 2016; Broom and Rychtář 2016). A large body of literature has analysed the number of equilibria, their stability and attainability in concrete strategic scenarios such as the public goods game and its variants, see for example Broom et al. (1997), Broom (2000), Pacheco et al. (2009), Souza et al. (2009), Peña (2012), Peña et al. (2014) and Sasaki et al. (2015). However, despite their importance, equilibrium properties in random games are far less understood with, to the best of our knowledge, only a few recent efforts (Gokhale and Traulsen 2010, 2014; Han et al. 2012; Galla and Farmer 2013; Duong and Han 2015, 2016; Broom and Rychtář 2016). One of the most challenging problems in the study of equilibrium properties in random games is to characterise the distribution of the number of equilibria (Gokhale and Traulsen 2010; Han et al. 2012):

What is the distribution of the number of (internal) equilibria in a d-player random evolutionary game and how can we compute it?

This question has been studied in the literature to some extent. For example, in Gokhale and Traulsen (2010, 2014) and Han et al. (2012), the authors studied this question with a small number of players ($d\le 4$) and only focused on the probability of attaining the maximal number of equilibrium points, i.e. $p_{d-1}$, where $p_m$ ($0\le m\le d-1$) is the probability that a *d*-player game with two strategies has exactly *m* internal equilibria. These works use a direct approach by analytically solving a polynomial equation, expressing the positivity of its zeros as domains of conditions for the coefficients and then integrating over these domains to obtain the corresponding probabilities. However, it is impossible to extend this approach to games with a large or arbitrary number of players as in general, a polynomial of degree five or higher is not analytically solvable (Abel 1824). In more recent works (Duong and Han 2015, 2016; Duong et al. 2017), we have established the links between random evolutionary games, random polynomial theory (Edelman and Kostlan 1995) and classical polynomial theory (particularly Legendre polynomials), employing techniques from the latter to study *the expected number* of internal equilibria, *E*. More specifically, we provided closed form formulas for *E*, characterised its asymptotic limits as the number of players in the game tends to infinity and investigated the effect of correlation in the case of correlated payoff entries. On the one hand, *E* offers useful information regarding the macroscopic, average behaviour of the number of internal equilibria a dynamical system might have. On the other hand, *E* cannot provide the level of complexity or the number of different states of biodiversity that will occur in the system. In these situations, details about how the number of internal equilibrium points distributed is required. Furthermore, as *E* can actually be derived from $p_m$ using the formula $E = \sum ^{d-1}_{m=0} m p_m$, a closed form formula for $p_m$ would make it possible to compute *E* for any *d*, hence filling in the gap in the literature on computing *E* for large *d* ($d\ge 5)$. Therefore, it is necessary to estimate $p_m$.

### Summary of main results

In this paper, we address the above question by providing a closed-form formula for the probability $p_m$ ($0\le m\le d-1$). Our approach is based on the links between random polynomial theory and random evolutionary game theory established in our previous work (Duong and Han 2015, 2016). That is, an internal equilibrium in a *d*-player game with two strategies can be found by solving the following polynomial equation (detailed derivation in Sect. 2),

1 $$\begin{aligned} \sum \limits _{k=0}^{d-1}\beta _k\begin{pmatrix} d-1\\ k \end{pmatrix} y^k=0, \end{aligned}$$

where $\beta _k=A_k-B_k$, with $A_k$ and $B_k$ being random variables representing the entries of the game payoff matrix. We now summarise the main results of this paper. Detailed derivations and proofs will be given in subsequent sections. The first main result is an explicit formula for the probability distribution of the number of internal equilibria.

#### Theorem 1

(The distribution of the number of internal equilibria in a *d*-player two-strategy random evolutionary game) Suppose that the coefficients $\{\beta _k\}$ in (1) are either normally distributed, uniformly distributed or the difference of uniformly distributed random variables. The probability that a *d*-player two-strategy random evolutionary game has *m*, $0\le m\le d-1$, internal equilibria, is given by

2 $$\begin{aligned} p_{m}=\sum _{k=0}^{\lfloor \frac{d-1-m}{2}\rfloor }p_{m,2k,d-1-m-2k}, \end{aligned}$$

where $p_{m,2k,d-1-m-2k}$ are given in (13), (14) and (15), respectively.

This theorem, which is stated in detail in Theorem 4 in Sect. 3, is derived from a more general theorem, Theorem 3, where we provide explicit formulas for the probability $p_{m,2k,n-m-2k}$ that a random polynomial of degree *n* has *m* ($0\le m\le n$) positive, 2*k* ($0\le k\le \lfloor \frac{n-m}{2}\rfloor$) complex and $n-2m-2k$ negative roots. Note that results from Theorem 3 are applicable to a wider class of general random polynomials, i.e. beyond those derived from evolutionary random games considered in this work.

Theorem 1 is theoretically interesting and can be used to compute $p_m$, $0\le m\le d-1$ for small *d*. We use it to compute all the probabilities $p_m$, $0\le m\le d-1$, for *d* up to 5, and compare the results with those obtained through extensive numerical simulations (for validation). However, when *d* is larger it becomes computationally expensive to compute these probabilities using formula (2) because one needs to calculate all the probabilities $p_{m,2k,d-1-2k}$, $0\le k\le \lfloor \frac{n-m}{2}\rfloor$, which are complex multiple integrals. To overcome this issue, in Sect. 5, we develop our second main result, Theorem 2 below, which offers simpler explicit estimates of $p_m$ in terms of *d* and *m*. The main idea in developing this result is employing the symmetry of the coefficients $\beta _k$. Specifically, we consider two cases

$$\begin{aligned}&\text {Case 1:}\quad \mathbf {P}(\beta _k>0)=\mathbf {P}(\beta _k<0)=\frac{1}{2},\\&\text {Case 2:}\quad \mathbf {P}(\beta _k>0)=\alpha \quad \text {and}\quad \mathbf {P}(\beta _k<0)=1-\alpha , \end{aligned}$$

for all $k=0,\ldots , d-1$ and for some $0\le \alpha \le 1$. Note here that Case 1 is an instance of Case 2 when $\alpha =\frac{1}{2}$ and can be satisfied when $a_k$ and $\beta _k$ are exchangeable (see Lemma 1 below). Interestingly, the symmetry of $\beta _k$ allows us to obtain a much simpler treatment. The general case allows us to move beyond the exchangeability condition capturing the fact that different strategies might have different payoff properties.

#### Theorem 2

We have the following upper-bound estimate for $p_m$

3 $$\begin{aligned} p_m\le \sum _{\begin{array}{c} k\ge m\\ k-m~\text {even} \end{array}} p_{k,d-1}, \end{aligned}$$

where $$p_{k,d-1}=\frac{1}{2^{d-1}}\begin{pmatrix} d-1\\ k \end{pmatrix}$$ if $\alpha =\frac{1}{2}$, in this case the sum on the right hand side of  (3) can be computed explicitly in terms of *m* and *d*. For the general case, it can be computed explicitly according to Theorem 7. The estimate (3) has several useful implications, leading to explicit bounds for $p_{d-2}$ and $p_{d-1}$ as well as the following assertions:

1. For $$d=2$$: $$p_0=\alpha ^2+(1-\alpha )^2$$ and $$p_1=2\alpha (1-\alpha )$$;
2. For $$d=3$$: $$p_1=2\alpha (1-\alpha )$$.

This theorem is a summary of Theorems 6, 7 and 8 in Sect. 4 that are derived using Descartes’ rule of signs and combinatorial methods. We note that results of the aforementioned theorems are applicable to a wider class of random polynomials that are not necessarily from random games.

### Organisation of the paper

The rest of the paper is organised as follows. In Sect. 2, we recall and summarise the replicator dynamics for multi-player two-strategy games. The main contributions of this paper and the detailed analysis of the main results described above will be presented in subsequent sections. Section 3 is devoted to the proof of Theorem 1 on the probability distribution. The proof of Theorem 2 will be given in Sect. 4. In Sect. 5 we show some numerical simulations to demonstrate analytical results. In Sect. 6, further discussions are given. Finally, Appendix 1 contains proofs of technical results from previous sections.

## Replicator dynamics

A fundamental model of evolutionary game theory is replicator dynamics (Taylor and Jonker 1978; Zeeman 1980; Hofbauer and Sigmund 1998; Schuster and Sigmund 1983; Nowak 2006), describing that whenever a strategy has a fitness larger than the average fitness of the population, it is expected to spread. For the sake of completeness, below we derive the replicator dynamics for multi-player two-strategy games.

Consider an infinitely large population with two strategies, A and B. Let *x*, $0 \le x \le 1$, be the frequency of strategy A. The frequency of strategy B is thus $(1-x)$. The interaction of the individuals in the population is in randomly selected groups of *d* participants, that is, they play and obtain their fitness from *d*-player games. The game is defined through a $(d-1)$-dimensional payoff matrix (Gokhale and Traulsen 2010), as follows. Let $A_k$ (respectively, $B_k$) be the payoff that an A-strategist (respectively, a B-strategist) obtained when playing with a group of $d-1$ players that consists of *k* A-strategists. In this paper, we consider symmetric games where the payoffs do not depend on the ordering of the players. Asymmetric games will be studied in a forthcoming paper. In the symmetric case, the probability that an A strategist interacts with *k* other A strategists in a group of size $d-1$ is

4 $$\begin{aligned} \begin{pmatrix} d-1\\ k \end{pmatrix}x^k (1-x)^{d-1-k}. \end{aligned}$$

Thus, the average payoffs of *A* and *B* are, respectively

$$\begin{aligned} \pi _A= \sum \limits _{k=0}^{d-1}A_k\begin{pmatrix} d-1\\ k \end{pmatrix}x^k (1-x)^{d-1-k}, \quad \pi _B = \sum \limits _{k=0}^{d-1}B_k\begin{pmatrix} d-1\\ k \end{pmatrix}x^k (1-x)^{d-1-k}. \end{aligned}$$

The replicator equation of a *d*-player two-strategy game is given by (Hofbauer and Sigmund 1998; Sigmund 2010; Gokhale and Traulsen 2010)

$$\begin{aligned} {\dot{x}}=x(1-x)\big (\pi _A-\pi _B\big ). \end{aligned}$$

Since $x=0$ and $x=1$ are two trivial equilibrium points, we focus only on internal ones, i.e. $0< x < 1$. They satisfy the condition that the fitnesses of both strategies are the same, i.e. $\pi _A=\pi _B$, which gives rise to

$$\begin{aligned} \sum \limits _{k=0}^{d-1}\beta _k \begin{pmatrix} d-1\\ k \end{pmatrix}x^k (1-x)^{d-1-k} = 0, \end{aligned}$$

where $\beta _k = A_k - B_k$. Using the transformation $y= \frac{x}{1-x}$, with $0< y < +\infty$, dividing the left hand side of the above equation by $(1-x)^{d-1}$ we obtain the following polynomial equation for *y*

5 $$\begin{aligned} P(y):=\sum \limits _{k=0}^{d-1}\beta _k\begin{pmatrix} d-1\\ k \end{pmatrix}y^k=0. \end{aligned}$$

Note that this equation can also be derived from the definition of an evolutionarily stable strategy (ESS), an important concept in EGT (Maynard Smith 1982), see e.g., Broom et al. (1997). Note however that, when moving to random evolutionary games with more than two strategies, the conditions for ESS are not the same as for those of stable equilibrium points of replicator dynamics. As in Gokhale and Traulsen (2010), Duong and Han (2015, 2016), we are interested in random games where $A_k$ and $B_k$ (thus $\beta _k$), for $0\le k\le d-1$, are random variables.

In Sect. 3 where we provide estimates for the number of internal equilibria in a *d*-player two-strategy game, we will use the information on the symmetry of $\beta _k$. The following lemma gives a necessary condition to determine when the difference of two random variables is symmetrically distributed.

### Lemma 1

(Duong et al. 2017, Lemma 3.5) Let *X* and *Y* be two exchangeable random variables, i.e. their joint probability distribution $f_{X,Y}(x,y)$ is symmetric, $f_{X,Y}(x,y)=f_{X,Y}(y,x)$. Then $Z=X-Y$ is symmetrically distributed about 0, i.e., its probability distribution satisfies $f_Z(z)=f_Z(-z)$. In addition, if *X* and *Y* are i.i.d then they are exchangeable.

For the sake of completeness, the proof of this Lemma is provided in Sect. 1.

## The distribution of the number of positive zeros of random polynomials and applications to EGT

This section focuses on deriving the distribution of the number of internal equilibria of a *d*-player two-strategy random evolutionary game. We recall that an internal equilibria is a real and positive zero of the polynomial *P*(*y*) in (5). We denote by $\kappa$ the number of positive zeros of this polynomial. For a given *m*, $0\le m\le d-1$, we need to compute the probability $p_m$ that $\kappa =m$. To this end, we first adapt a method introduced in Zaporozhets (2006) (see also Butez and Zeitouni 2017; Götze et al. 2017 for its applications to other problems) to establish a formula to compute the probability that a general random polynomial has a given number of real and positive zeros. Then we apply the general theory to the polynomial *P*.

### The distribution of the number of positive zeros of a random polynomial

Consider a general random polynomial

6 $$\begin{aligned} \mathbf {P}(t)=\xi _0 t^n+\xi _1t^{n-1}+\cdots +\xi _{n-1}t+\xi _n. \end{aligned}$$

We use the following notations for the elementary symmetric polynomials

7 $$\begin{aligned} \sigma _0(y_1,\ldots ,y_n)&=1,\nonumber \\ \sigma _1(y_1,\ldots ,y_n)&=y_1+\cdots +y_n,\nonumber \\ \sigma _2(y_1,\ldots ,y_n)&=y_1y_2+\cdots +y_{n-1}y_n,\nonumber \\&\vdots \nonumber \\ \sigma _{n-1}(y_1,\ldots ,y_n)&=y_1y_2\ldots y_{n-1}+\cdots +y_2y_3\ldots y_n,\nonumber \\ \sigma _{n}(y_1,\ldots ,y_n)&=y_1\ldots y_n, \end{aligned}$$

and denote by

8 $$\begin{aligned} \varDelta (y_1,\ldots ,y_n)=\prod _{1\le i<j\le n}|y_i-y_j| \end{aligned}$$

the Vandermonde determinant.

#### Theorem 3

Assume that the random variables $\xi _0,\xi _1,\ldots , \xi _n$ have a joint density $p(a_0,\ldots ,a_n)$. Let $0\le m\le d-1$ and $0\le k\le \lfloor \frac{n-m}{2}\rfloor$. The probability $p_{m,2k,n-m-2k}$ that $\mathbf {P}$ has *m* positive, 2*k* complex and $n-m-2k$ negative zeros is given by

9 $$\begin{aligned}&p_{m,2k,n-m-2k}=\frac{2^{k}}{m! k! (n-m-2k)!}\int _{\mathbf { R}_+^m}\int _{\mathbf { R}_-^{n-m-2k}} \int _{\mathbf { R}_+^k}\int _{[0,\pi ]^k}\int _{\mathbf { R}}\nonumber \\&\quad r_1\ldots r_k p(a\sigma _0,\ldots ,a\sigma _{n}) |a^{n}\varDelta |\, da\,d\alpha _1\ldots d\alpha _k dr_1\ldots dr_k dx_1\ldots dx_{n-2k},\qquad \end{aligned}$$

where

10 $$\begin{aligned}&\sigma _j=\sigma _j\left( x_1,\ldots ,x_{n-2k}, r_1e^{i\alpha _1}, r_1e^{-i\alpha _1},\ldots ,r_k e^{i\alpha _k}, r_k e^{-i \alpha _k}\right) , \end{aligned}$$

11 $$\begin{aligned}&\varDelta =\varDelta \left( x_1,\ldots ,x_{n-2k}, r_1e^{i\alpha _1}, r_1e^{-i\alpha _1},\ldots ,r_k e^{i\alpha _k}, r_k e^{-i \alpha _k}\right) . \end{aligned}$$

As consequences,

1. The probability that $\mathbf {P}$ has *m* positive zeros is
  $$\begin{aligned} p_{m}=\sum _{k=0}^{\lfloor \frac{n-m}{2}\rfloor }p_{m,2k,n-m-2k}. \end{aligned}$$
2. In particular, the probability that $\mathbf {P}$ has the maximal number of positive zeros is
  $$\begin{aligned} p_{n}=\frac{2^{k}}{k! (n-2k)!}\int _{\mathbf { R}_+^{n}}\int _{\mathbf { R}}p(a\sigma _0,\ldots ,a\sigma _{n})\, |a^{n}\,\varDelta |\, dadx_1\ldots dx_{n}, \end{aligned}$$
  where
  $$\begin{aligned} \sigma _j=\sigma _j(x_1,\ldots ,x_{n}),\quad \varDelta =\varDelta (x_1,\ldots ,x_{n}). \end{aligned}$$

#### Proof

The reference (Zaporozhets 2006, Theorem 1) provides a formula to compute the probability that the polynomial $\mathbf {P}$ has $n-2k$ real and 2*k* complex roots. In the present paper, we need to distinguish between positive and negative real zeros. We now sketch and adapt the proof of Theorem 1 of Zaporozhets (2006) to obtain the formula (9) for the probability that the polynomial $\mathbf {P}$ has *m* positive, 2*k* complex and $n-m-2k$ negative roots. Consider a $(n+1)$-dimensional vector space $\mathbf {V}$ of polynomials of the form

$$\begin{aligned} Q(t)=a_0t^n+a_1t^{n-1}+\cdots +a_{n-1}t+a_n, \end{aligned}$$

and a measure $\mu$ on this space defined as the integral of the differential form

12 $$\begin{aligned} dQ=p(a_0,\ldots ,a_n)\,da_0\wedge \cdots \wedge da_n. \end{aligned}$$

Our goal is to find $\mu (V_{m,2k})$ where $V_{m,2k}$ is the set of polynomials having *m* positive, 2*k* complex and $n-m-2k$ negative roots. Let $Q\in V_{m,2k}$. Denote all zeros of *Q* as

$$\begin{aligned}&z_1=x_1,\ldots ,z_{n-2k}=x_{n-2k},\quad z_{n-2k+1}=r_1 e^{i \alpha _1},\quad z_{n-2k+2}=r_1 e^{-i \alpha _1},\ldots ,\\&\quad z_{n-1}=r_k e^{i \alpha _k},\quad z_{n}=r_k e^{-i \alpha _k}, \end{aligned}$$

where

$$\begin{aligned}&0<x_1,\ldots , x_m<\infty ;\quad -\infty<x_{m+1},\ldots , x_{n-2k}<0; \quad 0<r_1,\ldots ,r_k<\infty ;\\&0<\alpha _1,\ldots ,\alpha _k<\pi . \end{aligned}$$

To find $\mu (V_{m,2k})$ we need to integrate the differential form (12) over the set $V_{m,2k}$. The key idea in the proof of Theorem 1 of Zaporozhets (2006) is to make a change of coordinates $(a_0,\ldots , a_n)\mapsto (a,x_1,\ldots ,x_{n-2k}, r_1,\ldots , r_k, \alpha _1,\ldots , \alpha _k)$, with $a=a_0$, and find *dQ* in the new coordinates. The derivation of the following formula is carried out in detail in Zaporozhets (2006):

$$\begin{aligned} dQ= & {} 2^k r_1\ldots r_k\, p\left( a,a\sigma _1\left( x_1,\ldots ,x_{n-2k},r_1e^{i\alpha _1},r_1e^{-i\alpha _1},\ldots ,r_ke^{i\alpha _k}, r_ke^{-i\alpha _k}\right) ,\right. \\&\left. \ldots a\sigma _n\left( x_1,\ldots ,x_{n-2k},r_1e^{i\alpha _1},r_1e^{-i\alpha _1},\ldots ,r_ke^{i\alpha _k}, r_ke^{-i\alpha _k}\right) \right) \\&\times \left| a^n \varDelta \left( \left( x_1,\ldots ,x_{n-2k},r_1e^{i\alpha _1},r_1e^{-i\alpha _1},\ldots ,r_ke^{i\alpha _k}, r_ke^{-i\alpha _k}\right) \right) \right| \\&\times \, dx_1\wedge \cdots \wedge dx_{n-2k}\wedge dr_1\wedge \cdots \wedge dr_k\wedge d\alpha _1\wedge \cdots \wedge d\alpha _k\wedge da. \end{aligned}$$

Now we integrate this equation over all polynomials *Q* that have *m* positive zeros, $n-m-2k$ negative zeros and *k* complex zeros in the upper half-plane. Since there are *m*! permutations of the positive zeros, $(n-m-2k)!$ permutations of the negative zeros, and *k*! permutations of the complex zeros, after integrating each polynomial in the left-hand side will occur $m!k!(n-m-2k)!$ times. Hence the integral of the left-hand side is equal to $m!k!(n-m-2k)! \, p_{m,2k,n-m-2k}$. The integral on the right-hand side equals

$$\begin{aligned}&2^k\int _{\mathbf { R}_+^m}\int _{\mathbf { R}_-^{n-m-2k}} \int _{\mathbf { R}_+^k}\int _{[0,\pi ]^k}\int _{\mathbf { R}} r_1\ldots r_k p(a\sigma _0,\ldots ,a\sigma _{n}) |a^{n}\varDelta |\, da\,d\alpha _1\ldots d\alpha _k\\&\quad dr_1\ldots dr_k dx_1\ldots dx_{n-2k}, \end{aligned}$$

hence the assertion (9) follows. $\square$

### The distribution of the number of internal equilibria

Next we apply Theorem 3 to compute the probability that a random evolutionary game has *m*, $0\le m\le d-1$, internal equilibria. We derive formulas for the three most common cases (Han et al. 2012):

1. $\{\beta _j,0\le j\le d-1\}$ are i.i.d. standard normally distributed,
2. $\{\beta _j\}$ are i.i.d. uniformly distributed with the common distribution $f_j(x)=\frac{1}{2} \mathbb {1}_{[-1,1]}(x)$,
3. $\{A_k\}$ and $\{B_k\}$ are i.i.d. uniformly distributed with the common distribution $f_j(x)=\frac{1}{2} \mathbb {1}_{[-1,1]}(x)$.

The main result of this section is the following theorem (cf. Theorem 2).

#### Theorem 4

The probability that a *d*-player two-strategy random evolutionary game has *m* ($0\le m\le d-1$) internal equilibria is

$$\begin{aligned} p_{m}=\sum _{k=0}^{\lfloor \frac{d-1-m}{2}\rfloor }p_{m,2k,d-1-m-2k}, \end{aligned}$$

where $p_{m,2k,d-1-m-2k}$ is given below for each of the cases above:

– For the case (C1)

13 $$\begin{aligned}&p_{m,2k,d-1-m-2k}\nonumber \\&\quad =\frac{2^{k}}{m! k! (d-1-m-2k)!}~\frac{ \varGamma \Big (\frac{d}{2}\Big ) }{(\pi )^{\frac{d}{2}}\prod \nolimits _{i=0}^{d-1}\delta _i} \int _{\mathbf { R}_+^m}\int _{\mathbf { R}_-^{d-1-2k-m}} \int _{\mathbf { R}_+^k}\int _{[0,\pi ]^k}\, r_1\ldots r_k\nonumber \\&\quad \quad \left( \sum \limits _{i=0}^{d-1}\frac{\sigma _i^2}{\delta _i^2}\right) ^{-\frac{d}{2}} \varDelta \,\, d\alpha _1\ldots d\alpha _k dr_1\ldots dr_k dx_1\ldots dx_{d-1-2k}, \end{aligned}$$

where $\sigma _i$, for $i=0,\ldots ,d-1$, and $\varDelta$ are given in (10)–(11) and $$\delta _i=\begin{pmatrix} d-1\\ i \end{pmatrix}$$.

– For the case (C2)

14 $$\begin{aligned}&p_{m,2k,d-1-m-2k}=\frac{2^{k+1-d}}{d \, m!\, k! \,(d-1-m-2k)!\prod \nolimits _{i=0}^{d-1} \delta _i}\int _{\mathbf { R}_+^m}\int _{\mathbf { R}_-^{d-1-2k-m}} \int _{\mathbf { R}_+^k}\int _{[0,\pi ]^k}\nonumber \\&\quad r_1\ldots r_k\,\Big (\min \big \{|\delta _i/\sigma _i|\big \}\Big )^{d} \varDelta \,\,d\alpha _1\ldots d\alpha _k dr_1\ldots dr_k dx_1\ldots dx_{d-1-2k}. \end{aligned}$$

– For the case (C3)

15 $$\begin{aligned}&p_{m,2k,d-1-m-2k}=\frac{2^{k+1}(-1)^d}{m! k! (d-1-m-2k)!\prod \nolimits _{j=0}^{d-1}\delta _j^2}\int _{\mathbf { R}_+^m}\int _{\mathbf { R}_-^{d-1-2k-m}} \int _{\mathbf { R}_+^k}\int _{[0,\pi ]^k}\nonumber \\&\quad r_1\ldots r_k\, \prod _{j=0}^{d-1}|\sigma _j|\sum _{i=0}^{d}(-1)^i \frac{K_i}{2d-i} \Big (\min \big \{|\delta _i/\sigma _i|\big \}\Big )^{2d-i}\varDelta \,d\alpha _1\ldots d\alpha _k dr_1\ldots dr_k \nonumber \\&\quad dx_1\ldots dx_{d-1-2k}. \end{aligned}$$

In particular, the probability that a *d*-player two-strategy random evolutionary game has the maximal number of internal equilibria is:

1. for the case (C1)
  16 $$\begin{aligned} p_{d-1}=\frac{1}{(d-1)!}~\frac{\varGamma \Big (\frac{d}{2}\Big ) }{(\pi )^\frac{d}{2} \prod \nolimits _{i=0}^{d-1}\delta _i}~\int _{\mathbf { R}_+^{d-1}} q(\sigma _0,\ldots ,\sigma _{d-1})\,dx_1\ldots dx_{d-1};\nonumber \\ \end{aligned}$$
2. for the case (C2)
  17 $$\begin{aligned} p_{d-1}=\frac{2^{1-d}}{d! \prod _{i=0}^{d-1} \delta _i}~\int _{\mathbf { R}_+^{d-1}}\Big (\min \big \{|\delta _i/\sigma _i|\big \}\Big )^{d} \varDelta \,dx_1\ldots dx_{d-1}; \end{aligned}$$
3. for the case (C3)
  18 $$\begin{aligned}&p_{d-1}=\frac{2(-1)^d}{(d-1)!\prod _{j=0}^{d-1}\delta _j^2}\int _{\mathbf { R}_+^{d-1}}\prod _{j=0}^{d-1}|\sigma _j|\sum _{i=0}^{d}(-1)^i \frac{K_i}{2d-i} \Big (\min \big \{|\delta _i/\sigma _i|\big \}\Big )^{2d-i}\varDelta \nonumber \\&\,dx_1\ldots dx_{d-1}. \end{aligned}$$

Note that in formulas (16)–(18) above

$$\begin{aligned} \sigma _j=\sigma _j(x_1,\ldots ,x_{d-1}),\quad \varDelta =\varDelta (x_1,\ldots ,x_{d-1}). \end{aligned}$$

#### Proof

(1) Since $\{\beta _j,0\le j\le d-1\}$ are i.i.d. standard normally distributed, the joint distribution $p(y_0,\ldots ,y_{d-1})$ of $$\left\{ \begin{pmatrix} d-1\\ j \end{pmatrix}\beta _j,0\le j\le d-1\right\} $$ is given by

$$\begin{aligned} p(y_0,\ldots ,y_{d-1})&=\frac{1}{(2\pi )^{\frac{d}{2}} \prod _{i=0}^{d-1}\begin{pmatrix} d-1\\ i \end{pmatrix}}\exp \left[ -\frac{1}{2}\sum _{i=0}^{d-1}\frac{y_i^2}{\begin{pmatrix} d-1\\ i \end{pmatrix}^2}\right] \\&=\frac{1}{(2\pi )^{\frac{d}{2}}|\mathcal { C}|^\frac{1}{2}}\exp \left[ -\frac{1}{2}\mathbf {y}^T\mathcal { C}^{-1}\mathbf {y}\right] , \end{aligned}$$

where $\mathbf {y}=[y_0~~y_1~~\ldots ~~y_{d-1}]^T$ and $\mathcal { C}$ is the covariance matrix

$$\begin{aligned} \mathcal { C}_{ij}=\begin{pmatrix} d-1\\ i \end{pmatrix}\begin{pmatrix} d-1\\ j \end{pmatrix}\delta _{ij}. \end{aligned}$$

Therefore,

19 $$\begin{aligned} p(a\sigma _0,\ldots , a \sigma _{d-1})=\frac{1}{(2\pi )^\frac{d}{2} |\mathcal { C}|^\frac{1}{2}}\exp \Bigg (-\frac{a^2}{2}\varvec{\sigma }^T\,\mathcal { C}^{-1}\,\varvec{\sigma }\Bigg ), \end{aligned}$$

where $\varvec{\sigma }=[\sigma _0~\sigma _1~\ldots ~\sigma _{d-1}]^T$. Using the following formula for moments of a normal distribution,

$$\begin{aligned} \int _{\mathbf { R}}|x|^n\exp \big (-\alpha x^2\big )\,dx=\frac{\varGamma \big (\frac{n+1}{2}\big )}{\alpha ^\frac{n+1}{2}}, \end{aligned}$$

we compute

$$\begin{aligned} \int _{\mathbf { R}}|a|^{d-1}\exp \Bigg (-\frac{a^2}{2}\varvec{\sigma }^T\,\mathcal { C}^{-1}\,\varvec{\sigma }\Bigg )\,da=\frac{\varGamma \Big (\frac{d}{2}\Big )}{\Big (\frac{\varvec{\sigma }^T\mathcal { C}^{-1} \varvec{\sigma }}{2}\Big )^{\frac{d}{2}}}=\frac{2^\frac{d}{2}\varGamma \Big (\frac{d}{2}\Big )}{ \big (\varvec{\sigma }^T\mathcal { C}^{-1}\varvec{\sigma }\big )^{\frac{d}{2}}}. \end{aligned}$$

Applying Theorem 3 to the polynomial *P* given in (5) and using the above identity we obtain

$$\begin{aligned}&p_{m,2k,d-1-m-2k}\\&\quad =\frac{2^{k}}{m! k! (d-1-m-2k)!}\int _{\mathbf { R}_+^m}\int _{\mathbf { R}_-^{d-1-2k-m}} \int _{\mathbf { R}_+^k}\int _{[0,\pi ]^k}\int _{\mathbf { R}}\\&\qquad \quad r_1\ldots r_k\, p(a\sigma _0,\ldots ,a\sigma _{d-1}) |a|^{d-1}\varDelta \, da\,d\alpha _1\ldots d\alpha _k dr_1\ldots dr_k dx_1\ldots dx_{d-1-2k}\\&\quad =\frac{2^{k}}{m! k! (d-1-m-2k)!}~\frac{1}{(2\pi )^\frac{d}{2} |\mathcal { C}|^\frac{1}{2}}~ 2^{\frac{d}{2}}\varGamma \Big (\frac{d}{2}\Big ) ~\int _{\mathbf { R}_+^m}\int _{\mathbf { R}_-^{d-1-2k-m}} \int _{\mathbf { R}_+^k}\int _{[0,\pi ]^k}\\&\qquad \quad r_1\ldots r_k\, \big (\varvec{\sigma }^T\mathcal { C}^{-1}\varvec{\sigma }\big )^{-\frac{d}{2}}~\varDelta \,d\alpha _1\ldots d\alpha _k dr_1\ldots dr_k dx_1\ldots dx_{d-1-2k}\\&\quad =\frac{2^{k}}{m! k! (d-1-m-2k)!}~\frac{\varGamma \Big (\frac{d}{2}\Big ) }{(\pi )^\frac{d}{2} |\mathcal { C}|^\frac{1}{2}}~\int _{\mathbf { R}_+^m}\int _{\mathbf { R}_-^{d-1-2k-m}} \int _{\mathbf { R}_+^k}\int _{[0,\pi ]^k}\\&\qquad \quad r_1\ldots r_k\, \big (\varvec{\sigma }^T\mathcal { C}^{-1}\varvec{\sigma }\big )^{-\frac{d}{2}}~\varDelta \,d\alpha _1\ldots d\alpha _k dr_1\ldots dr_k dx_1\ldots dx_{d-1-2k}, \end{aligned}$$

which is the desired equality (13) by definition of $\mathcal { C}$ and $\varvec{\sigma }$.

(2) Now since $\{\beta _j\}$ are i.i.d. uniformly distributed with the common distribution $f_j(x)=\frac{1}{2} \mathbb {1}_{[-1,1]}(x)$, the joint distribution $p(y_0,\ldots ,y_{d-1})$ of

$$\begin{aligned} \left\{ \begin{pmatrix} d-1\\ j \end{pmatrix}\beta _j,0\le j\le d-1\right\} \end{aligned}$$

is given by

$$\begin{aligned} p(y_0,\ldots ,y_{d-1})=\frac{1}{2^{d}\prod _{i=0}^{d-1} \delta _i}\mathbb {1}_{\times _{i=0}^{d-1}[-\delta _i,\delta _i]}(y_0,\ldots , y_{d-1}) \quad \text {where } \delta _{i}= \begin{pmatrix} d-1\\ i \end{pmatrix}. \end{aligned}$$

Therefore,

$$\begin{aligned} p(a\sigma _0,\ldots ,a \sigma _{d-1})=\frac{1}{2^{d}\prod _{i=0}^{d-1} \delta _i}\mathbb {1}_{\times _{i=0}^{d-1}[-\delta _i,\delta _i]}(a\sigma _0,\ldots , a \sigma _{d-1}). \end{aligned}$$

Since $\mathbb {1}_{\times _{i=0}^{d-1}[-\delta _i,\delta _i]}(a\sigma _0,\ldots , a \sigma _{d-1})=1$ if and only if $a\sigma _i\in [-\delta _i,\delta _i]$ for all $i=0,\ldots , d-1$, i.e., if and only if

$$\begin{aligned} a\in \bigcap \limits _{i=0}^{d-1} \big [-|\delta _i/\sigma _i|,|\delta _i/\sigma _i|\big ]=\left[ -\min \limits _{i \in \{0,\ldots , d-1\}}\big \{|\delta _i/\sigma _i|\big \},\min \limits _{i \in \{0,\ldots , d-1\}}\big \{|\delta _i/\sigma _i|\big \}\right] , \end{aligned}$$

we have (for simplicity of notation, in the subsequent computations we shorten $\min \nolimits _{i \in \{0,\ldots , d-1\}}$ by $\min$)

$$\begin{aligned} p(a\sigma _0,\ldots ,a \sigma _{d-1})= {\left\{ \begin{array}{ll}\frac{1}{2^{d}\prod _{i=0}^{d-1} \delta _i},&{}\text {if } a \in \big [-\min \big \{|\delta _i/\sigma _i|\big \},\min \big \{|\delta _i/\sigma _i|\big \}\big ],\\ 0, &{}\text {otherwise}. \end{array}\right. } \end{aligned}$$

Therefore,

$$\begin{aligned} \int _{\mathbf { R}}|a|^{d-1}p(a\sigma _0,\ldots ,a \sigma _{d-1})\,da&=\frac{1}{2^{d}\prod _{i=0}^{d-1} \delta _i} \int _{-\min \big \{|\delta _i/\sigma _i|\big \}}^{\min \big \{|\delta _i/\sigma _i| \big \}}|a|^{d-1}\,da\\&=\frac{1}{d\, 2^{d-1}\prod _{i=0}^{d-1} \delta _i} \Big (\min \big \{|\delta _i/ \sigma _i|\big \}\Big )^{d}. \end{aligned}$$

Similarly as in the normal case, using this identity and applying Theorem 3 we obtain

$$\begin{aligned}&p_{m,2k,d-1-m-2k}\\&\quad =\frac{2^{k}}{m! k! (d-1-m-2k)!}\int _{\mathbf { R}_+^m}\int _{\mathbf { R}_-^{d-1-2k-m}} \int _{\mathbf { R}_+^k}\int _{[0,\pi ]^k}\int _{\mathbf { R}}\\&\qquad \quad r_1\ldots r_k\, p(a\sigma _0,\ldots ,a\sigma _{d-1}) |a|^{d-1}\varDelta \, da\,d\alpha _1\ldots d\alpha _k dr_1\ldots dr_k dx_1\ldots dx_{d-1-2k}\\&\quad =\frac{2^{k+1-d}}{d \, m!\, k! \,(d-1-m-2k)! \prod _{i=0}^{d-1} \delta _i}\int _{\mathbf { R}_+^m}\int _{\mathbf { R}_-^{d-1-2k-m}} \int _{\mathbf { R}_+^k}\int _{[0,\pi ]^k}\\&\qquad \quad r_1\ldots r_k\, \Big (\min \big \{|\delta _i/\sigma _i|\big \}\Big )^{d} \varDelta \, da\,d\alpha _1\ldots d\alpha _k dr_1\ldots dr_k dx_1\ldots dx_{d-1-2k}. \end{aligned}$$

(3) Now we assume that $A_j$ and $B_j$ are i.i.d. uniformly distributed with the common distribution $\gamma (x)=\frac{1}{2} \mathbb {1}_{[-1,1]}(x)$. Since $\beta _j=A_j-B_j$, its probability density is given by

$$\begin{aligned} \gamma _{\beta }(x)=\int _{-\infty }^{+\infty }f(y)f(x+y)\,dy=(1-|x|) \mathbb {1}_{[}-1,1](x). \end{aligned}$$

The probability density of $\delta _j\beta _j$ is

$$\begin{aligned} \gamma _{j}(x)=\frac{1}{\delta _j}\left( 1-\frac{|x|}{\delta _j}\right) \mathbb {1}_{[-1,1]}(x/\delta _j)=\frac{\delta _j-|x|}{\delta _j^2}\mathbb {1}_{[- \delta _j,\delta _j]}(x), \end{aligned}$$

and the joint distribution $p(y_0,\ldots ,y_{d-1})$ of $\left\{ \delta _j\beta _j,0\le j\le d-1\right\}$ is given by

$$\begin{aligned} p(y_0,\ldots ,y_{d-1})=\prod _{j=0}^{d-1}\frac{\delta _j-|y_j|}{\delta _j^2} \mathbb {1}_{\times _{i=0}^{d-1}[-\delta _i,\delta _i]}(y_0,\ldots , y_{d-1}). \end{aligned}$$

Therefore

$$\begin{aligned} p(a\sigma _0,\ldots ,a \sigma _{d-1})=\prod \limits _{j=0}^{d-1}\frac{\delta _j-|a \sigma _j|}{\delta _j^2}\mathbb {1}_{\times _{i=0}^{d-1}[-\delta _i,\delta _i]}( a \sigma _0,\ldots ,a \sigma _{d-1}). \end{aligned}$$

We compute

$$\begin{aligned}&\int _{\mathbf { R}}|a|^{d-1}p(a\sigma _0,\ldots ,a \sigma _{d-1})\,da\\&\quad =\frac{1}{\prod _{j=0}^{d-1}\delta _j^2}\int _{-\min \big \{|\delta _i/\sigma _i|\big \}}^{\min \big \{|\delta _i/\sigma _i|\big \}}|a|^{d-1}\prod _{j=0}^{d-1}(\delta _j-|a\sigma _j|)\,da\\&\quad =\frac{2}{\prod _{j=0}^{d-1}\delta _j^2}\int _{0}^{\min \big \{|\delta _i/\sigma _i|\big \}}a^{d-1}\prod _{j=0}^{d-1}(\delta _j-a|\sigma _j|)\,da\\&\quad =2 (-1)^d \prod _{j=0}^{d-1}\frac{|\sigma _j|}{\delta _j^2}\int _{0}^{\min \big \{|\delta _i/\sigma _i|\big \}}a^{d-1}\prod _{j=0}^{d-1}\left( a-\frac{\delta _j}{|\sigma _j|}\right) \,da\\&\quad =2 (-1)^d \prod _{j=0}^{d-1}\frac{|\sigma _j|}{\delta _j^2}\sum _{i=0}^{d}(-1)^i K_i\int _{0}^{\min \big \{|\delta _i/\sigma _i|\big \}}a^{2d-1-i}\,da\\&\quad =2 (-1)^d \prod _{j=0}^{d-1}\frac{|\sigma _j|}{\delta _j^2}\sum _{i=0}^{d}(-1)^i \frac{K_i}{2d-i} \Big (\min \big \{|\delta _i/\sigma _i|\big \}\Big )^{2d-i}, \end{aligned}$$

where $$K_i=\sigma _i(\delta _0/|\sigma _0|,\ldots , \delta _{d-1}/|\sigma _{d-1}|)$$ for $$i=0,\ldots , d$$.

Therefore,

$$\begin{aligned}&p_{m,2k,d-1-m-2k}\\&\quad =\frac{2^{k}}{m! k! (d-1-m-2k)!}\int _{\mathbf { R}_+^m}\int _{\mathbf { R}_-^{d-1-2k-m}} \int _{\mathbf { R}_+^k}\int _{[0,\pi ]^k}\int _{\mathbf { R}}\\&\qquad \quad r_1\ldots r_k\, p(a\sigma _0,\ldots ,a\sigma _{d-1}) |a|^{d-1}\varDelta \, da\,d\alpha _1\ldots d\alpha _k dr_1\ldots dr_k dx_1\ldots dx_{d-1-2k}\\&\quad =\frac{2^{k+1}(-1)^d}{m! k! (d-1-m-2k)!\prod _{j=0}^{d-1}\delta _j^2}\int _{\mathbf { R}_+^m}\int _{\mathbf { R}_-^{d-1-2k-m}}\int _{\mathbf { R}_+^k}\int _{[0,\pi ]^k}\\&\qquad \quad r_1\ldots r_k\, \prod _{j=0}^{d-1}|\sigma _j|\sum _{i=0}^{d}(-1)^i \frac{K_i}{2d-i} \Big (\min \big \{|\delta _i/\sigma _i|\big \}\Big )^{2d-i}\varDelta \,d\alpha _1\ldots d\alpha _k dr_1\ldots dr_k\\&\qquad \quad dx_1\ldots dx_{d-1-2k}. \end{aligned}$$

$$\square $$

#### Corollary 1

The expected numbers of internal equilibria and stable internal equilibria, *E*(*d*) and *SE*(*d*), respectively, of a *d*-player two-strategy game, are given by

$$\begin{aligned} E(d)=\sum _{m=0}^{d-1} m p_m, \quad \quad SE(d)=\frac{1}{2}\sum _{m=0}^{d-1} m p_m. \end{aligned}$$

Note that this formula for *E*(*d*) is applicable for non-normal distributions, which is in contrast to the method used in previous works (Duong and Han 2015, 2016) that can only be used for normal distributions. The second part, i.e. the formula for the expected number of stable equilibrium points, was obtained based on the following property of stable equilibria in multi-player two-strategy evolutionary games, as shown in Han et al. (2012, Theorem 3): $SE(d) = \frac{1}{2}E(d)$.

#### Remark 1

In Theorem 4 for the case (C1), the assumption that $\beta _k$’s are standard normal distributions, i.e. having variance 1, is just for simplicity. Suppose that $\beta _k$’s are normal distributions with mean 0 and variance $\eta ^2$. We show that the probability $p_{m}$, for $0\le m\le d-1$, does not depend on $\eta$. In this case, the formula for *p* is given by (19) but with $\mathcal { C}$ being replaced by $\eta ^2 \mathcal { C}$. To indicate its dependence on $\eta$, we write $p_\eta$. We use a change of variable $a=\eta {\tilde{a}}$. Then

$$\begin{aligned}&a^{d-1}p_\eta (a\sigma _0,\ldots , a\sigma _{d-1})\,da\\&\quad =\eta ^{d-1}{\tilde{a}}^{d-1}\frac{1}{(\sqrt{2\pi }\eta )^{d}\prod _{j=0}^{d-1} \begin{pmatrix} d-1\\ j \end{pmatrix}}\exp \left[ -\frac{{\tilde{a}}^2}{2}\sum _{j=0}^{d-1}\frac{\sigma _j^2}{\begin{pmatrix} d-1\\ j \end{pmatrix}^2}\right] \eta \, d{\tilde{a}}\\&\quad ={\tilde{a}}^{d-1}\frac{1}{(\sqrt{2\pi })^{d}\prod _{j=0}^{d-1} \begin{pmatrix} d-1\\ j \end{pmatrix}}\exp \left[ -\frac{{\tilde{a}}^2}{2}\sum _{j=0}^{d-1}\frac{\sigma _j^2}{\begin{pmatrix} d-1\\ j \end{pmatrix}^2}\right] \, d{\tilde{a}}\\&\quad ={\tilde{a}}^{d-1}p_1({\tilde{a}}\sigma _0,\ldots , {\tilde{a}}\sigma _{d-1}), \end{aligned}$$

from which we deduce that $p_m$ does not depend on $\eta$. Similarly for the other cases, the uniform interval can be $\frac{1}{2\alpha }[-\alpha ,\alpha ]$ for some $\alpha >0$.

**Fig. 1:**
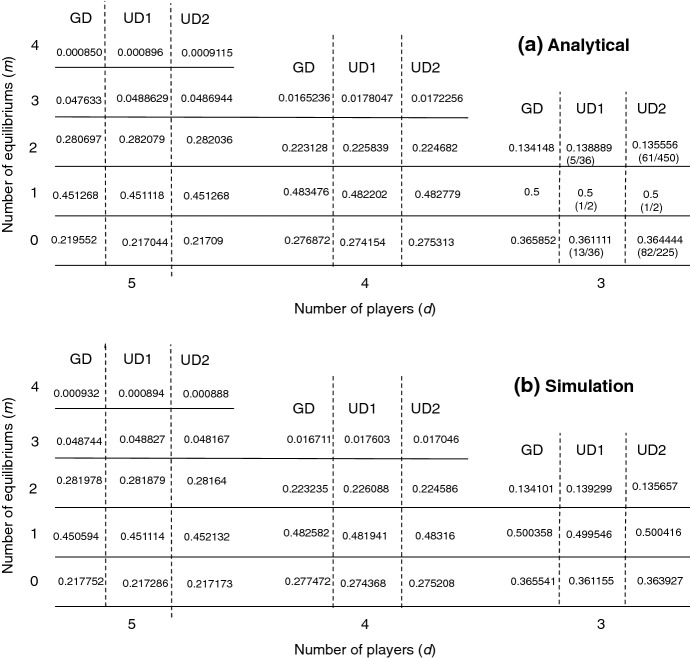
Numerical versus simulation calculations of the probability of having a concrete number (*m*) of internal equilibria, $p_m$, for different values of *d*. The payoff entries $a_k$ and $b_k$ were drawn from a normal distribution with variance 1 and mean 0 (GD) and from a standard uniform distribution (UD2). We also study the case where $\beta _k = a_k - b_k$ itself is drawn from a standard uniform distribution (UD1). Results are obtained from analytical formulas (Theorem 2) (**a**) and are based on sampling $10^6$ payoff matrices (**b**) where payoff entries are drawn from the corresponding distributions. Analytical and simulations results are in accordance with each other. All results are obtained using Mathematica

For illustration of the application of Theorem 4, the following examples show explicit calculations for $d=3$ and 4 for the case of normal distributions, i.e. (C1). Further numerical results for $d = 5$ and also for other distributions, i.e. (C2) and (C3), are provided in Fig. 1. The integrals in these examples were computed using Mathematica.

### Examples for $d=3,4$

#### Example 1

(*Three-player two-strategy games*: $d = 3$) (1) One internal equilibria: $p_1=p_{1,0,1}$. We have

$$\begin{aligned}&m = 1, \quad k = 0,\quad \sigma _0=1, \quad \sigma _1=x_1+x_2,\quad \sigma _2= x_1x_2,\quad \varDelta =|x_2-x_1|,\\&q(\sigma _0,\sigma _1,\sigma _2)=\frac{1}{\left( 1+x_1^2 x_2^2+\frac{1}{4} \left( x_1+x_2\right) {}^2\right) {}^{3/2}} |x_2-x_1|. \end{aligned}$$

Substituting these values into (13) we obtain the probability that a three-player two-strategy evolutionary game has 1 internal equilibria

$$\begin{aligned} p_{1}=\frac{1}{4 \pi }\int _{\mathbf { R}_+}\int _{\mathbf { R}_-}\frac{1}{\left( 1+x_1^2 x_2^2+\frac{1}{4} \left( x_1+x_2\right) {}^2\right) {}^{3/2}} |x_2-x_1| \,dx_1\,dx_2 = 0.5. \end{aligned}$$

(2) Two internal equilibria: $p_2=p_{2,0,0}$. We have

$$\begin{aligned}&m = 2, \quad k = 0,\quad \sigma _0=1,\quad \quad \sigma _1=x_1+x_2,\quad \sigma _2= x_1x_2,\quad \varDelta =|x_2-x_1|,\\&q(\sigma _0,\sigma _1,\sigma _2)=\frac{1}{\left( 1+x_1^2 x_2^2+\frac{1}{4} \left( x_1+x_2\right) {}^2\right) {}^{3/2}}|x_2-x_1|. \end{aligned}$$

The probability that a three-player two-strategy evolutionary game has 2 internal equilibria is

20 $$\begin{aligned} p_2=\frac{1}{8\pi }\int _{\mathbf { R}_+^2}\frac{1}{\left( 1+x_1^2 x_2^2+\frac{1}{4} \left( x_1+x_2\right) {}^2\right) {}^{3/2}} |x_2-x_1| \,dx_1\,dx_2 \ \approx 0.134148.\nonumber \\ \end{aligned}$$

(3) No internal equilibria: the probability that a three-player two-strategy evolutionary game has no internal equilibria is $p_0=1-p_1-p_2 \ \approx 1 - 0.5 - 0.134148 = 0.365852.$

#### Example 2

(*Four-player two-strategy games*: $d=4$)

(1) One internal equilibria: $p_{1}=p_{1,0,2}+p_{1,2,0}$.

We first compute $p_{1,0,2}$. In this case,

$$\begin{aligned}&m=1,\quad k=0,\quad \sigma _0=1, \quad \sigma _1=x_1+x_2+x_3,\quad \sigma _2= x_1x_2+x_1x_3+x_2x_3,\\&\varDelta =|x_2-x_1|\, |x_3-x_1|\,|x_3-x_2|. \end{aligned}$$

Substituting these into (13) we get

$$\begin{aligned} p_{1,0,2}= & {} \frac{1}{18\pi ^2}\int _{\mathbf { R}_-}\int _{\mathbf { R}_-}\int _{\mathbf { R}_+} \left( 1+\frac{(x_1+x_2+x_3)^2}{9}+\frac{(x_1x_2+x_1x_3+x_2x_3)^2}{9}+(x_1x_2x_3)^2 \right) ^{-2}\\&\times |x_2-x_1|\,|x_3-x_1|\, |x_3-x_2|\,dx_1\,dx_2\,dx_3 \ \approx 0.223128. \end{aligned}$$

Next we compute $p_{1,2,0}$. In this case,

$$\begin{aligned}&m=1, \quad k=1,\quad \sigma _0=1,\\&\sigma _1=\sigma _1\left( x_1,r_1e^{i\alpha _1}, r_1 e^{-i\alpha _1}\right) =x_1+r_1e^{i\alpha _1}+ r_1 e^{-i\alpha _1}=x_1+2r_1\cos \left( \alpha _1\right) , \\&\sigma _2=\sigma _2\left( x_1,r_1e^{i\alpha _1}, r_1 e^{-i\alpha _1}\right) =x_1\left( r_1e^{i\alpha _1}+r_1e^{-i\alpha _1}\right) +r_1^2=2x_1r_1\cos \left( \alpha _1\right) +r_1^2,\\&\sigma _3=\sigma _3\left( x_1,r_1e^{i\alpha _1}, r_1 e^{-i\alpha _1}\right) =x_1r_1^2,\\&\varDelta =\varDelta \left( x_1,r_1e^{i\alpha _1}, r_1 e^{-i\alpha _1}\right) =\left| r_1e^{i\alpha _1}-x_1\right| \left| r_1e^{-i\alpha _1}-x_1\right| \left| r_1e^{i\alpha _1}-r_1e^{-i\alpha _1}\right| \\&~~~=\left| r_1^2-2x_1r_1\cos \left( \alpha _1\right) +x_1^2\right| \left| 2r_1\sin \left( \alpha _1\right) \right| . \end{aligned}$$

Substituting these into (13) yields

$$\begin{aligned} p_{1,2,0}= & {} \frac{2}{9\pi ^2} \int _{\mathbf { R}_+}\int _{[0,\pi ]}\int _{\mathbf { R}_+}r_1\, \left( 1+\frac{(x_1+2r_1\cos (\alpha _1))^2}{9}+\frac{(2x_1r_1\cos (\alpha _1)+r_1^2)^2}{9}+(x_1r_1^2)^2 \right) ^{-2}\\&\times \, |r_1^2-2x_1r_1\cos (\alpha _1)+x_1^2||2r_1\sin (\alpha _1)|\,dx_1dr_1d\alpha _1da \ \approx 0.260348. \end{aligned}$$

Therefore, we obtain that

$$\begin{aligned} p_{1}=p_{1,0,2}+p_{1,2,0}\ \approx 0.223128 +0.260348 = 0.483476. \end{aligned}$$

(2) Two internal equilibria: $p_2=p_{2,0,1}$

$$\begin{aligned}&m=2, \quad k=0, \quad \sigma _0=1,\quad \sigma _1=x_1+x_2+x_3, \quad \sigma _2=x_1x_2+x_1x_3+x_2x_3,\\&\quad \sigma _3=x_1x_2x_3, \varDelta =|x_2-x_1|\,|x_3-x_1|\, |x_3-x_2|. \end{aligned}$$

The probability that a four-player two-strategy evolutionary game has 2 internal equilibria is

21 $$\begin{aligned} p_2= & {} \frac{1}{18\pi ^2}\int _{\mathbf { R}_+}\int _{\mathbf { R}_+}\int _{\mathbf { R}_-} \left( 1+\frac{(x_1+x_2+x_3)^2}{9}+\frac{(x_1x_2+x_1x_3+x_2x_3)^2}{9}+(x_1x_2x_3)^2\right) ^{-2}\nonumber \\&\times \, |x_2-x_1|\,|x_3-x_1|\, |x_3-x_2|\,dx_1\,dx_2\,dx_3 \ \approx 0.223128. \end{aligned}$$

(3) Three internal equilibria: $p_3=p_{3,0,0}$

$$\begin{aligned}&m=3,\quad k=0, \quad \sigma _0=1,\quad \sigma _1=x_1+x_2+x_3, \quad \sigma _2=x_1x_2+x_1x_3+x_2x_3,\\&\quad \sigma _3=x_1x_2x_3,\quad \varDelta =|x_2-x_1|\,|x_3-x_1|\, |x_3-x_2|. \end{aligned}$$

The probability that a four-player two-strategy evolutionary game has 3 internal equilibria is

$$\begin{aligned} p_3&=\frac{1}{54\pi ^2}\int _{\mathbf { R}_+^3} \left( 1+\frac{(x_1+x_2+x_3)^2}{9}+\frac{(x_1x_2+x_1x_3+x_2x_3)^2}{9}+(x_1x_2x_3)^2\right) ^{-2}\\&\quad \times |x_2-x_1|\,|x_3-x_1|\, |x_3-x_2|\,dx_1\,dx_2\,dx_3 \ \approx 0.0165236. \end{aligned}$$

(4) No internal equilibria: the probability that a four-player two-strategy evolutionary game has no internal equilibria is: $p_0=1-p_1-p_2-p_3 \ \approx 1 - 0.483476 - 0.223128 - 0.0165236 = 0.276872$.

## Universal estimates for $p_m$

In Sect. 3, we have derived closed-form formulas for the probability distributions $p_m \ (0\le m\le d-1)$ of the number of internal equilibria. However, it is computationally expensive to compute these probabilities since it involves complex multiple-dimensional integrals. In this section, using Descartes’ rule of signs and combinatorial techniques, we provide universal estimates for $p_m$. Descartes’ rule of signs is a technique for determining an upper bound on the number of positive real roots of a polynomial in terms of the number of sign changes in the sequence formed by its coefficients. This rule has been applied to random polynomials before in the literature (Bloch and Pólya 1932); however this paper only obtained estimates for the expected number of zeros of a random polynomial.

### Theorem 5

(Descartes’ rule of signs, see e.g., Curtiss 1918) Consider a polynomial of degree *n*, $p(x)=a_nx^n+\cdots +a_0$ with $a_n\ne 0$. Let *v* be the number of variations in the sign of the coefficients $a_n,a_{n-1},\ldots ,a_0$ and $n_p$ be the number of real positive zeros. Then $(v-n_p)$ is an even non-negative integer.

We recall that an internal equilibrium of a *d*-player two-strategy game is a positive root of the polynomial *P* given in (5). We will apply Descartes’ rule of signs to find an upper bound for the probability that a random polynomial has a certain number of positive roots. This is a problem that is of interest in its own right and may have applications elsewhere; therefore we will first study this problem for a general random polynomial of the form

22 $$\begin{aligned} p(y):=\sum _{k=0}^n a_k y^k, \end{aligned}$$

and then apply it to the polynomial *P*. It turns out that the symmetry of $\{a_k\}$ will be the key: the asymmetric case requires completely different treatment from the symmetric one.

### Estimates of $p_m$: symmetric case

We first consider the case where the coefficients $\{a_k\}$ in (22) are symmetrically distributed. The main result of this section will be Theorem 6 that provides several upper and lower bounds for the probability that a *d*-player two strategy game has *m* internal equilibria. Before stating Theorem 6, we need the following auxiliary lemmas.

#### Proposition 1

Suppose that the coefficients $a_k, 0\le k\le n$ in the polynomial (22) are i.i.d. and symmetrically distributed. Let $p_{k,n}, 0\le k\le n$, be the probability that the sequence of coefficients $(a_0,\ldots ,a_{n})$ has *k* changes of signs. Then

23 $$\begin{aligned} p_{k,n}=\frac{1}{2^{n}}\begin{pmatrix} n\\ k \end{pmatrix}. \end{aligned}$$

#### Proof

See Appendix 2. $$\square $$

The next two lemmas on the sum of binomial coefficients will be used later on.

#### Lemma 2

Let $0\le k \le n$ be positive integers. Then it holds that

$$\begin{aligned}&\sum _{\begin{array}{c} j=k\\ j:\text {even} \end{array}}^{n}\begin{pmatrix} n\\ j \end{pmatrix}=\frac{1}{2}\left[ \sum _{j=0}^{n-k}\begin{pmatrix} n\\ j \end{pmatrix}+(-1)^{k}\begin{pmatrix} n-1\\ k-1 \end{pmatrix}\right] , \\&\sum _{\begin{array}{c} j=k\\ j:\text {odd} \end{array}}^{n}\begin{pmatrix} n\\ j \end{pmatrix}=\frac{1}{2}\left[ \sum _{j=0}^{n-k}\begin{pmatrix} n\\ j \end{pmatrix}-(-1)^{k}\begin{pmatrix} n-1\\ k-1 \end{pmatrix}\right] , \end{aligned}$$

where it is understood that $$\begin{pmatrix} n\\ j \end{pmatrix}=0$$ if $j<0$. In particular, for $k=0$, we get

24 $$\begin{aligned} \sum _{\begin{array}{c} j=0\\ j:\text {even} \end{array}}^{n}\begin{pmatrix} n\\ j \end{pmatrix}=\sum _{\begin{array}{c} j=0\\ j:\text {odd} \end{array}}^{n}\begin{pmatrix} n\\ j \end{pmatrix}=2^{n-1}. \end{aligned}$$

#### Proof

See Appendix 3. $$\square $$

The following lemma provides estimates on the sum of the first *k* binomial coefficients.

#### Lemma 3

Let *n* and $0\le k\le n$ be positive integers. We have the following estimates (MacWilliams and Sloane 1977, Lemma 8 and Corollary 9, Chapter 10; Gottlieb et al. 2012)

25 $$\begin{aligned}&\frac{2^{nH\big (\frac{k}{n}\big )}}{\sqrt{8k\big (1-\frac{k}{n}\big )}}\le \sum _{j=0}^k\begin{pmatrix} n\\ j \end{pmatrix}\le \delta 2^{nH\big (\frac{k}{n}\big )}\quad \text {if }0\le k< \frac{n}{2},\quad {and} \end{aligned}$$

26 $$\begin{aligned}&2^n-\delta 2^{nH\big (\frac{k}{n}\big )}\le \sum _{j=0}^k\begin{pmatrix} n\\ j \end{pmatrix}\le 2^n-\frac{2^{nH\big (\frac{k}{n}\big )}}{\sqrt{8k\big (1-\frac{k}{n} \big )}}\quad \text {if } \frac{n}{2}\le k\le n, \end{aligned}$$

where $\delta =0.98$ and *H* is the binary entropy function

27 $$\begin{aligned} H(x)=-x\log _2(x)-(1-x)\log _2(1-x), \end{aligned}$$

where $0\log _2 0$ is taken to be 0. In addition, if $n=2n'$ is even and $0\le k\le n'$, we also have the following estimate (Lovász et al. 2003, Lemma 3.8.2)

28 $$\begin{aligned} \sum _{j=0}^{k-1}\begin{pmatrix} 2n'\\ j \end{pmatrix}\le 2^{2n'-1}\begin{pmatrix} 2n'\\ k \end{pmatrix}\Big /\begin{pmatrix} 2n'\\ n' \end{pmatrix}. \end{aligned}$$

We now apply Proposition 1 and Lemmas 2 and 3 to derive estimates for the probability that a *d*-player two-strategy evolutionary game has a certain number of internal equilibria. The main theorem of this section is the following.

#### Theorem 6

Suppose that the coefficients $\{\beta _k\}$ in (5) are symmetrically distributed. Let $p_m, 0\le m\le d-1,$ be the probability that the *d*-player two-strategy random game has *m* internal equilibria. Then the following assertions hold

- Upper-bound for $p_m$, for all $0\le m\le d-1$,
  29 $$\begin{aligned} p_m&\le \frac{1}{2^{d-1}}\sum \limits _{\begin{array}{c} j: j\ge m \\ j-m~\text {even} \end{array}}\begin{pmatrix} d-1\\ j \end{pmatrix}=\frac{1}{2^d}\left[ \sum _{j=0}^{d-1-m}\begin{pmatrix} d-1\\ j \end{pmatrix}+\begin{pmatrix} d-2\\ m-1 \end{pmatrix}\right] \end{aligned}$$
  30 $$\begin{aligned}&\le {\left\{ \begin{array}{ll} \frac{1}{2^d}\left[ \delta 2^{(d-1)H\big (\frac{m}{d-1}\big )}+\begin{pmatrix} d-2\\ m-1 \end{pmatrix}\right] &{}\text {if}~~\frac{d-1}{2}< m\le d-1,\\ \frac{1}{2^d}\Bigg [2^{d-1}-\frac{2^{(d-1)H\big (\frac{m}{d-1}\big )}}{8m \big (1-\frac{m}{d-1}\big )} +\begin{pmatrix} d-2\\ m-1 \end{pmatrix}\Bigg ]&\text {if}~~0\le m\le \frac{d-1}{2}. \end{array}\right. } \end{aligned}$$
  As consequences, $0\le p_m\le \frac{1}{2}$ for all $0\le m\le d-1$, $p_{d-1}\le \frac{1}{2^{d-1}}$, $p_{d-2}\le \frac{d-1}{2^{d-1}}$ and $\lim \nolimits _{d\rightarrow \infty }p_{d-1}=\lim \nolimits _{d\rightarrow \infty }p_{d-2}=0$. In addition, if $d-1=2 d'$ is even and $0\le m\le d'$ then
  31 $$\begin{aligned} p_m\le \frac{1}{2^d}\left[ 2^{d-2}\begin{pmatrix} d-1\\ m-1 \end{pmatrix}\Big /\begin{pmatrix} d-1\\ d' \end{pmatrix}+\begin{pmatrix} d-2\\ m-1 \end{pmatrix}\right] . \end{aligned}$$
- Lower-bound for $p_0$ and $p_1$:
  32 $$\begin{aligned} p_0\ge \frac{1}{2^{d-1}}\quad {and}\quad p_1\ge \frac{d-1}{2^{d-1}}. \end{aligned}$$
- For $$d=2$$: $$p_0=p_1=\frac{1}{2}$$.
- For $$d=3$$: $$p_1=\frac{1}{2}$$.

#### Proof

(a) This part is a combination of Decartes’ rule of signs, Proposition 1 and Lemmas 2 and 3. In fact, as a consequence of this rule and by Proposition 1, we have

$$\begin{aligned} p_m\le \sum _{\begin{array}{c} j: j\ge m\\ j-m:~\text {even} \end{array}}p_{j,d-1}=\frac{1}{2^{d-1}}\sum _{\begin{array}{c} j: j\ge m\\ j-m:~\text {even} \end{array}}\begin{pmatrix} d-1\\ j \end{pmatrix}, \end{aligned}$$

which is the inequality part in (29). Next, applying Lemma 2 for $k=m$ and $n=d-1$ and then Lemma 3, we obtain

$$\begin{aligned}&\frac{1}{2^{d-1}}\sum _{\begin{array}{c} k: k\ge m\\ k-m:~ \text {even} \end{array}}\begin{pmatrix} d-1\\ k \end{pmatrix}\\&\quad = {\left\{ \begin{array}{ll}\frac{1}{2^{d}}\Bigg [ \sum \nolimits _{j=0}^{d-1-m}\begin{pmatrix} d-1\\ j \end{pmatrix}+(-1)^m\begin{pmatrix} d-2\\ m-1 \end{pmatrix}\Bigg ]&{}\text {if}~m~\text { is even} \\ \frac{1}{2^{d}}\Bigg [ \sum \nolimits _{j=0}^{d-1-m}\begin{pmatrix} d-1\\ j \end{pmatrix}-(-1)^m\begin{pmatrix} d-2\\ m-1 \end{pmatrix}\Bigg ]&\text {if}~m~\text { is odd} \end{array}\right. }\\&\quad =\frac{1}{2^d}\Bigg [ \sum \nolimits _{j=0}^{d-1-m}\begin{pmatrix} d-1\\ j \end{pmatrix}+\begin{pmatrix} d-2\\ m-1 \end{pmatrix}\Bigg ]\\&\quad \le {\left\{ \begin{array}{ll} \frac{1}{2^d}\Bigg [ \delta 2^{(d-1)H\big (\frac{m}{d-1}\big )}+\begin{pmatrix} d-2\\ m-1 \end{pmatrix}\Bigg ]&{}\text {if}~~\frac{d-1}{2} < m\le d-1,\\ \frac{1}{2^d}\Bigg [2^{d-1}-\frac{2^{(d-1)H\big (\frac{m}{d-1}\big )}}{8m \big (1-\frac{m}{d-1}\big )} +\begin{pmatrix} d-2\\ m-1 \end{pmatrix}\Bigg ]&\text {if}~~0\le m\le \frac{d-1}{2}. \end{array}\right. } \end{aligned}$$

This proves the equality part in (29) and (30). As a result, the estimate $p_m\le \frac{1}{2}$ for all $0\le m\le d-1$ is followed from (29) and (24); the estimates $p_{d-1}\le \frac{1}{2^{d-1}}$ and $p_{d-2}\le \frac{d-1}{2^{d-1}}$ are special cases of (29) for $m=d-1$ and $m=d-2$, respectively.

Finally, the estimate (31) is a consequence of (29) and (28).

(b) It follows from Decartes’ rule of signs and Proposition 1 that

$$\begin{aligned} p_0\ge p_{0,d-1}=\frac{1}{2^{d-1}}\quad \text {and}\quad p_{1}\ge p_{1,d-1}=\frac{d-1}{2^{d-1}}. \end{aligned}$$

(c) For $d=2$: from parts (a) and (b) we have

$$\begin{aligned} \frac{1}{2}\le p_0,\quad p_1\le \frac{1}{2}, \end{aligned}$$

which implies that $p_0=p_1=\frac{1}{2}$ as claimed.

(d) Finally, for $d=3$: also from parts (a) and (b) we get

$$\begin{aligned} \frac{1}{2}\le p_1\le \frac{1}{2}, \end{aligned}$$

so $p_1=\frac{1}{2}$. This finishes the proof of Theorem 6. $\square$

#### Remark 2

**Fig. 2:**
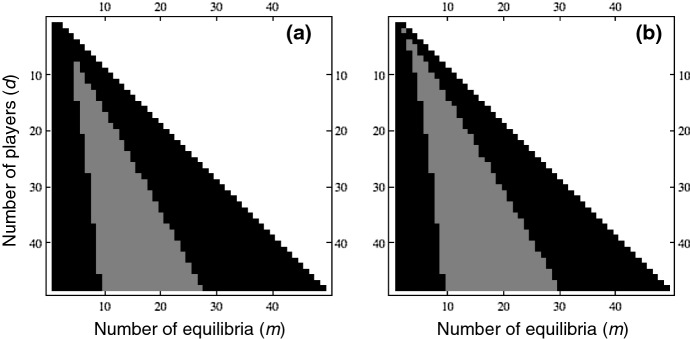
Comparison of the new upper bounds of $p_m$ derived in Theorem 6 with that of *E*(*d*) / *m*: **a** for the bound in (36) and **b** for the bound in (37). Black areas indicate when the former ones are better and the grey areas otherwise. Clearly the bound in (**a**) is stricter/better than that of (**b**). For small *d*, the new bounds are better. When *d* is sufficiently large, we observe that for any *d*, the new bounds are worse than *E*(*d*) / *m* when *m* is intermediate while better otherwise. Overall, this comparison indicates which formulas should be used to obtain a stricter upper bound of $p_m$

Note that in Theorem 6 we only assume that $\beta _k$ are symmetrically distributed but do not require that they are normal distributions. When $\{\beta _k\}$ are normal distributions, we have derived (Duong and Han 2015, 2016) a closed formula for the expected number *E*(*d*) of internal equilibria, which can be computed efficiently for large *d*. Since $E(d)=\sum _{m=0}^{d-1}m p_m$, we have $p_m\le E(d)/m$ for all $1\le m\le d-1$. Therefore, when $\{\beta _k\}$ are normal, we obtain an upper bound for $p_m$ as the minimum between *E*(*d*) / *m* and the bound obtained in Theorem 6. The comparison of the new bounds with *E*(*d*) / *m* in Fig. 2 shows that the new ones do better for *m* closer to 0 or $d-1$ but worse for intermediate *m* (i.e. closer to $(d-1)/2$).

### Estimates of $p_m$: general case

In the proof of Proposition 1 the assumption that $\{a_k\}$ are symmetrically distributed is crucial. In that case, all the $2^n$ binary sequences constructed are equally distributed, resulting in a compact formula for $p_{k,n}$. However, when $\{a_k\}$ are not symmetrically distributed, those binary sequences are no longer equally distributed. Thus computing $p_{k,n}$ becomes much more intricate. We now consider the general case where

$$\begin{aligned} \mathbf {P}(a_i>0)=\alpha ,~~\mathbf {P}(a_i<0)=1-\alpha \quad \text {for all}~ i=0,\ldots ,n. \end{aligned}$$

Note that the general case allows us to move beyond the usual assumption in the analysis of random evolutionary games that all payoff entries $a_k$’s and $b_k$’s have the same probability distribution resulting in $\alpha = 1/2$ (see Lemma 1). In the general case it only requires that all $a_k$’s have the same distribution and all $b_k$’s have the same distribution, capturing the fact that different strategies, i.e. *A* and *B* in Sect. 2, might have different payoff properties (e.g., defectors always have a larger payoff than cooperators in a public goods game).

The main results of this section will be Theorem 7 and Theorem 8. The former provides explicit formulas for $p_{k,n}$ while the latter consists of several upper and lower bounds for $p_m$. We will need several technically auxiliary lemmas whose proofs will be given in Appendix 1. We start with the following proposition that provides explicit formulas for $p_{k,n}$ for $k\in \{0,1,n-1,n\}$.

#### Proposition 2

The following formulas hold:

$$\begin{aligned}&\bullet \quad p_{0,n}=\alpha ^{n+1}+(1-\alpha )^{n+1},\quad p_{1,n}={\left\{ \begin{array}{ll} \frac{n}{2^n}&{}\text {if}~\alpha =\frac{1}{2},\\ 2\alpha (1-\alpha )\frac{(1-\alpha )^n-\alpha ^n}{1-2\alpha }&{}\text {if}~\alpha \ne \frac{1}{2}; \end{array}\right. } \\&\bullet \quad p_{n-1,n}={\left\{ \begin{array}{ll} n \alpha ^\frac{n}{2}(1-\alpha )^\frac{n}{2}&{}\text {if } n \text { even},\\ \alpha ^\frac{n+1}{2}(1-\alpha )^\frac{n+1}{2}\bigg [\frac{n+1}{2}\Big (\frac{\alpha }{1- \alpha }+\frac{1-\alpha }{\alpha }\Big )+(n-1)\bigg ]&{}\text {if }n \text { odd}; \end{array}\right. } \\&\bullet \quad p_{n,n}={\left\{ \begin{array}{ll} \alpha ^{\frac{n}{2}}(1-\alpha )^{\frac{n}{2}}&{}\text {if } n \text { is even},\\ 2 \alpha ^\frac{n+1}{2}(1-\alpha )^\frac{n+1}{2}&{}\text {if } n \text { is odd}. \end{array}\right. } \end{aligned}$$

In particular, if $\alpha =\frac{1}{2}$, then $p_{0,n}=p_{1,n}=\frac{1}{2^n}\text { and } p_{1,n}=p_{n-1,n}=\frac{n}{2^n}$.

#### Proof

See Appendix 4. $$\square $$

The computations of $p_{k,n}$ for other *k* are more involved. We will employ combinatorial techniques and derive recursive formulas for $p_{k,n}$. We define

$$\begin{aligned}&u_{k,n}=\mathbf {P}(\text {there are } k \text { variations of signs in}~\{a_0,\ldots ,a_n\}\big \vert a_{n}>0), \\&v_{k,n}=\mathbf {P}(\text {there are } k \text { variations of signs in}~\{a_0,\ldots ,a_n\}\big \vert a_{n}<0). \end{aligned}$$

We have the following lemma.

#### Lemma 4

The following recursive relations hold:

33 $$\begin{aligned} u_{k,n}=\alpha u_{k,n-1}+(1-\alpha )v_{k-1,n-1} \quad \text {and}\quad v_{k,n}=\alpha u_{k-1,n-1}+(1-\alpha )v_{k,n-1}.\qquad \end{aligned}$$

#### Proof

See Appendix 5. $$\square $$

We can decouple the recursive relations in Lemma 4 to obtain recursive relations for $\{u_{k,n}\}$ and $v_{k,n}$ separately as follows:

#### Lemma 5

The following recursive relations hold

$$\begin{aligned}&u_{k,n} =\alpha (1-\alpha )(u_{k-2,n-2}-u_{k,n-2})+u_{k,n-1},\\&v_{k,n} =\alpha (1-\alpha )(v_{k-2,n-2}-v_{k,n-2})+v_{k,n-1}. \end{aligned}$$

#### Proof

See Appendix 6. $$\square $$

Using the recursive equations for $u_{k,n}$ and $v_{k,n}$ we can also derive a recursive relation for $p_{k,n}$.

#### Proposition 3

$\{p_{k,n}\}$ satisfies the following recursive relation.

34 $$\begin{aligned} p_{k,n}=\alpha (1-\alpha )(p_{k-2,n-2}-p_{k,n-2})+p_{k,n-1}. \end{aligned}$$

#### Proof

See Appendix 7. $$\square $$

#### Remark 3

Proposition 3 provides a second-order recursive relation for the probabilities $\{p_{k,n}\}$. This relation resembles the well-known Chu–Vandermonde identity for binomial coefficients, $$\Big \{b_{k,n}:=\begin{pmatrix} n\\ k \end{pmatrix}\Big \}$$, which is that, for $0<m<n$,

$$\begin{aligned} b_{k,n}=\sum \limits _{j=0}^k \begin{pmatrix} m\\ j \end{pmatrix}b_{k-j,n-m}. \end{aligned}$$

Particularly for $m=2$ we obtain

$$\begin{aligned} b_{k,n}&=b_{k,n-2}+2b_{k-1,n-2}+b_{k-2,n-2}\\&=b_{k-2,n-2}-b_{k,n-2}+2(b_{k,n-2}+b_{k-1,n-2})\\&=b_{k-2,n-2}-b_{k,n-2}+2b_{k,n-1}, \end{aligned}$$

where the last identity is Pascal’ rule for binomial coefficients.

On the other hand, the recursive formula $p_{k,n}$ for $\alpha =\frac{1}{2}$ becomes

$$\begin{aligned} p_{k,n}=\frac{1}{4}(p_{k-2,n-2}-p_{k,n-2})+p_{k,n-1}. \end{aligned}$$

Using the transformation $a_{k,n}:=\frac{1}{2^n}p_{k,n}$ as in the proof of Theorem 7, then

$$\begin{aligned} a_{k,n}=a_{k-2,n-2}-a_{k,n-2}+2a_{k,n-1}, \end{aligned}$$

which is exactly the Chu–Vandermonde identity for $m=2$ above. Then it is no surprise that in Theorem 7 we obtain that $a_{k,n}$ is exactly the same as the binomial coefficient $$a_{k,n}=\begin{pmatrix} n\\ k \end{pmatrix}$$.

In the next main theorem we will find explicit formulas for $\{p_{k,n}\}$ from the recursive formula in the previous lemma using the method of generating functions. The case $\alpha =\frac{1}{2}$ will be a special one.

#### Theorem 7

$p_{k,n}$ is given explicitly by: for $\alpha =\frac{1}{2}$,

$$\begin{aligned} p_{k,n}=\frac{1}{2^n}\begin{pmatrix} n\\ k \end{pmatrix}. \end{aligned}$$

For $$\alpha \ne \frac{1}{2}$$:

(i) if *k* is even, $k=2k'$, then

$$\begin{aligned} p_{k,n}={\left\{ \begin{array}{ll} \sum \nolimits _{m=\lceil \frac{n}{2}\rceil }^n \frac{n-k+1}{2m-n+1}\begin{pmatrix} m\\ k',n-k'-m,2m-n \end{pmatrix}(-1)^{n-k'-m}(\alpha (1-\alpha ))^{n-m}&{}\\ &{}\text {if } n \text { even},\\ \sum \nolimits _{m=\lceil \frac{n}{2}\rceil }^n \frac{n-k+1}{2m-n+1}\begin{pmatrix} m\\ k',n-k'-m,2m-n \end{pmatrix}(-1)^{n-k'-m}(\alpha (1-\alpha ))^{n-m}&{}\\ \quad +\,2\begin{pmatrix} \lceil \frac{n-1}{2}\rceil \\ k' \end{pmatrix} (-1)^{\lceil \frac{n-1}{2}\rceil -k'+1} (\alpha (1-\alpha ))^{\frac{n+1}{2}}&\text {if } n \text { odd}; \end{array}\right. } \end{aligned}$$

(ii) if *k* is odd, $k=2k'+1$, then

$$\begin{aligned} p_{k,n}=2\,\sum _{m=\lceil \frac{n-1}{2}\rceil }^n\begin{pmatrix} m\\ k', n-k'-m-1,2m-n+1 \end{pmatrix}(-1)^{n-k'-m-1} (\alpha (1-\alpha ))^{n-m}. \end{aligned}$$

#### Proof

See Appendix 8. $$\square $$

#### Example 3

Below we provide explicit formulas for $\{p_{k,n}\}$ for $0\le k\le n\le 4$:

$$\begin{aligned} \bullet \quad n=1{:}&\quad p_{0,1}=\alpha ^2+(1-\alpha )^2; \quad p_{1,1}=2\alpha (1-\alpha );\\ \bullet \quad n=2{:}&\quad p_{0,2}=\alpha ^3+(1-\alpha )^3, \quad p_{1,2}=2\alpha (1-\alpha ),\quad p_{2,2}=\alpha (1-\alpha );\\ \bullet \quad n=3{:}&\quad p_{0,3}=\alpha ^4+(1-\alpha )^4,~~p_{1,3}=2\alpha (1-\alpha )(\alpha ^2-\alpha +1),\\&\quad p_{2,3}=2\alpha (1-\alpha )(\alpha ^2-\alpha +1), \quad p_{3,3}=2\alpha ^2(1-\alpha )^2;\\ \bullet \quad n=4{:}&\quad p_{0,4}=\alpha ^5+(1-\alpha )^5,~~p_{1,4}=2\alpha (1-\alpha )(2\alpha ^2-2\alpha +1),\\&\quad p_{2,4}=3\alpha (1-\alpha )(2\alpha ^2-2\alpha +1),\quad p_{3,4}=4\alpha ^2(1-\alpha )^2,~~p_{4,4}=\alpha ^2(1-\alpha )^2. \end{aligned}$$

Direct computations verify the recursive formula for $k=2,n=4$

$$\begin{aligned} p_{2,4}=\alpha (1-\alpha )(p_{0,2}-p_{2,2})+p_{2,3}. \end{aligned}$$

We now apply Theorem 7 to the polynomial *P* in (5) to obtain estimates for $p_m, 0\le m\le d-1$, which is the probability that a *d*-player two-strategy random evolutionary game has *m* internal equilibria. This theorem extends Theorem 6 for $\alpha =1/2$ to the general case although we do not achieve an explicit upper bound in terms of *d* as in Theorem 6.

#### Theorem 8

The following assertions hold

(i) Upper-bound for $p_m$
  $$\begin{aligned} p_m\le \sum _{\begin{array}{c} k\ge m\\ k-m~\text {even} \end{array}} p_{k,d-1}, \end{aligned}$$
  where $p_{k,d-1}$ can be computed explicitly according to Theorem 7 with *n* replaced by $d-1$.
(ii) Lower-bound for $p_0$: $p_0\ge \alpha ^{d}+(1-\alpha )^{d}\ge \frac{1}{2^{d-1}}$.
(iii) Lower-bound for $p_1$: $$p_1\ge {\left\{ \begin{array}{ll} \frac{d-1}{2^{d-1}}&{}\text {if}~\alpha =\frac{1}{2},\\ 2\alpha (1-\alpha )\frac{(1-\alpha )^{d-1}-\alpha ^{d-1}}{1-2\alpha } &{}\text {if}~\alpha \ne \frac{1}{2}. \end{array}\right. }$$
(iv) Upper-bound for $p_{d-2}$:
  $$\begin{aligned} p_{d-2}&\le {\left\{ \begin{array}{ll} (d-1) \alpha ^\frac{d-1}{2}(1-\alpha )^\frac{d-1}{2}&{}\text {if } d \text { odd},\\ \alpha ^\frac{d}{2}(1-\alpha )^\frac{d}{2}\bigg [\frac{d}{2}\Big (\frac{\alpha }{1-\alpha }+ \frac{1-\alpha }{\alpha }\Big )+(d-2)\bigg ]&{}\text {if } d \text { even},\end{array}\right. }\nonumber \\&\le \frac{d-1}{2^{d-1}}\quad \text {when}~d\ge 3. \end{aligned}$$
(v) Upper-bound for $p_{d-1}$:
  $$\begin{aligned} q_{d-1}&\le {\left\{ \begin{array}{ll} \alpha ^{\frac{d-1}{2}}(1-\alpha )^{\frac{d-1}{2}}&{}\text {if } d \text { is odd},\\ 2 \alpha ^\frac{d}{2}(1-\alpha )^\frac{d}{2}&{}\text {if } d \text { is even}, \end{array}\right. }\\&\le \frac{1}{2^{d-1}}. \end{aligned}$$

As consequences:

- For $$d=2$$: $$p_0=\alpha ^2+(1-\alpha )^2$$ and $$p_1=2\alpha (1-\alpha )$$.
- For $$d=3$$, $$p_1=2\alpha (1-\alpha )$$.

#### Proof

We will apply Decartes’ rule of signs, Proposition 2 and Theorem 7 for the random polynomial (5). It follows from Decartes’ rule of signs that

$$\begin{aligned} p_m\le \sum _{\begin{array}{c} k\ge m\\ k-m~\text {even} \end{array}} p_{k,d-1}, \end{aligned}$$

where $p_{k,d-1}$ is given explicitly in Theorem 7 with *n* replaced by $d-1$. This proves the first statement. In addition, we can also deduce from Decartes’ rule of signs and Proposition 2 the following estimates for special cases $m\in \{0,1,d-2,d-1\}$:

$$\begin{aligned}&\bullet ~~p_0\ge p_{0,d-1}=\alpha ^d+(1-\alpha )^d\ge \min _{0\le \alpha \le 1}[\alpha ^d+(1-\alpha )^d]=\frac{1}{2^{d-1}};\\&\bullet ~~ p_1\ge p_{1,d-1}={\left\{ \begin{array}{ll} \frac{d-1}{2^{d-1}}&{}\text {if}~\alpha =\frac{1}{2},\\ 2\alpha (1-\alpha )\frac{(1-\alpha )^{d-1}-\alpha ^{d-1}}{1-2\alpha }&{} \text {if}~\alpha \ne \frac{1}{2}; \end{array}\right. }\\&\bullet ~~ p_{d-2}\le p_{d-2,d-1}={\left\{ \begin{array}{ll} (d-1) \alpha ^\frac{d-1}{2}(1-\alpha )^\frac{d-1}{2}&{}\text {if } d \text { odd},\\ \alpha ^\frac{d}{2}(1-\alpha )^\frac{d}{2}\bigg [\frac{d}{2}\Big (\frac{\alpha }{ 1-\alpha }+\frac{1-\alpha }{\alpha }\Big )+(d-2)\bigg ]&{}\text {if } d \text { even}, \end{array}\right. }\\&\quad \quad \quad \quad \quad \quad \quad \quad \quad \quad = {\left\{ \begin{array}{ll} (d-1) (\alpha (1-\alpha ))^\frac{d-1}{2}&{}\text {if } d \text { odd},\\ \frac{d}{2}(\alpha (1-\alpha ))^{d/2-1}-2(\alpha (1-\alpha ))^{d/2}&{}\text {if } d \text { even}, \end{array}\right. } \\&\quad \quad \quad \quad \quad \quad \quad \quad \quad \quad \quad \le {\left\{ \begin{array}{ll} (d-1)(1/4)^\frac{d-1}{2}=\frac{d-1}{2^{d-1}}&{}\text {if } d \text { odd},\\ \max _{0\le \beta \le \frac{1}{4}} f(\beta )=\frac{d-1}{2^{d-1}}&{}\text {if } d\ge 3 \text { even}; \end{array}\right. }\\&\text {where}, \beta \!:=\!\alpha (1-\alpha ),~~ f(\beta ):=\frac{d}{2}\beta ^{d/2-1}-2\beta ^{d/2}, \text { and to obtain the last inequality}\\&\text {we have used the fact that}~~0\le \beta =\alpha (1-\alpha )\le \frac{1}{4}~\text {and}\\&f'(\beta )=d\beta ^{d/2-2}\Big (\frac{d}{4}-\frac{1}{2}-\beta \Big )\ge 0~~\text {when}~~0\le \beta \le \frac{1}{4}~~\text {and}~~ d\ge 3.\\&\bullet ~~ p_{d-1}\le p_{d-1,d-1}={\left\{ \begin{array}{ll} \alpha ^{\frac{d-1}{2}}(1-\alpha )^{\frac{d-1}{2}}&{}\text {if } d \text { is odd},\\ 2 \alpha ^\frac{d}{2}(1-\alpha )^\frac{d}{2}&{}\text {if } d \text { is even}, \end{array}\right. }\\&\quad \quad \quad \quad \quad \quad \quad \quad \quad \quad \quad \le {\left\{ \begin{array}{ll} (1/4)^\frac{d-1}{2}=\frac{1}{2^{d-1}}&{}\text {if } d \text { is odd},\\ 2(1/4)^\frac{d}{2}=\frac{1}{2^{d-1}} &{}\text {if } d \text { is even}. \end{array}\right. } \end{aligned}$$

These computations establish the estimates (*ii*)–(*v*) of the theorem. For the consequences: for $d=2$, in this case the above estimates (*ii*)–(*v*) respectively become:

$$\begin{aligned}&p_0\ge \alpha ^2+(1-\alpha )^2, \quad p_1\ge {\left\{ \begin{array}{ll} \frac{1}{2}&{}\text {if}~ \alpha =\frac{1}{2},\\ 2\alpha (1-\alpha )&{}\text {if}~ \alpha \ne \frac{1}{2} \end{array}\right. }=2\alpha (1-\alpha ),\quad \text {and}\\&p_0\le \alpha (1-\alpha )\Big [\frac{\alpha }{1-\alpha }+\frac{1-\alpha }{\alpha }\Big ]=\alpha ^2+(1-\alpha )^2, \quad q_1\le 2\alpha (1-\alpha ), \end{aligned}$$

which imply that $p_0=\alpha ^2+(1-\alpha )^2,\quad p_1=2\alpha (1-\alpha )$.

Similarly for $d=3$, estimates (*ii*) and (*iii*) respectively become

$$\begin{aligned} p_1\ge {\left\{ \begin{array}{ll} \frac{1}{2}\quad \text {if}~~\alpha =\frac{1}{2},\\ 2\alpha (1-\alpha )\text {if}~~\alpha \ne \frac{1}{2} \end{array}\right. }=2\alpha (1-\alpha ),\quad \text {and}\quad p_1\le 2\alpha (1-\alpha ), \end{aligned}$$

from which we deduce that $p_1=2\alpha (1-\alpha )$. $\square$

## Numerical simulations

In this section, we perform several numerical (sampling) simulations and calculations to illustrate the analytical results obtained in previous sections. Figure 1 shows the values of $\{p_m\}$ for $d\in \{3,4,5\}$, for the three cases studied in Theorem 4, i.e., when $\beta _k$ are i.i.d. standard normally distributed (GD), uniformly distributed (UD1) and when $\beta _k=a_k-b_k$ with $a_k$ and $\beta _k$ being uniformly distributed (UD2). We compare results obtained from analytical formulas in Theorem 4 and from samplings. The figure shows that they are in accordance with each other agreeing to at least 2 digits after the decimal points. Figure 2 compares the new upper bound obtained in Theorem 6 with that of *E*(*d*) / *m*. The comparison indicates which formulas should be used to obtain a stricter upper bound of $p_m$.

## Further discussions and future research

In this paper, we have provided closed-form formulas and universal estimates for the probability distribution of the number of internal equilibria in a *d*-player two-strategy random evolutionary game. We have explored further connections between evolutionary game theory and random polynomial theory as discovered in our previous works (Duong and Han 2015, 2016; Duong et al. 2017). We believe that the results reported in the present work open up a new exciting avenue of research in the study of equilibrium properties of random evolutionary games. We now provide further discussions on these issues and possible directions for future research.

*Computations of probabilities*$\{p_m\}$. Although we have found analytical formulas for $p_m$ it is computationally challenging to deal with them because of their complexity. Obtaining an effective computational method for $\{p_m\}$ would be an interesting problem for future investigation.

*Quantification of errors in the mean-field approximation theory* (Schehr and Majumdar 2008). Consider a general polynomial $\mathbf {P}$ as given in (6) with dependent coefficients, and let $P_m([a,b],n)$ be the probability that $\mathbf {P}$ has *m* real roots in the interval [*a*, *b*] (recall that *n* is the degree of the polynomial, which is equal to $d -1$ in Equation (1)). The mean-field theory (Schehr and Majumdar 2008) neglects the correlations between the real roots and simply considers that these roots are randomly and independently distributed on the real axis with some local density *f*(*t*) at point *t*, with *f*(*t*) being the density that can be computed from the Edelman–Kostlan theorem (Edelman and Kostlan 1995). Within this approximation in the large *n* limit, the probability $P_m([a, b],n)$ is given by a non-homogeneous Poisson distribution, see Schehr and Majumdar (2008, Section 3.2.2 and Equation (70)). By applying the mean-field theory one can approximate the probability $p_m$ that a random *d*-player two-strategy evolutionary game has *m* internal equilibria by a simpler and computationally feasible formula. However, it is unclear to us how to quantify the errors of approximation. We leave this topic for future research.

*Extensions to multi-strategy games*. We have focused in this paper on random games with two strategies (with an arbitrary number of players). The analysis of games with more than two strategies is much more intricate since in this case one needs to deal with systems of multi-variate random polynomials. We have provided (Duong and Han 2015, 2016) a closed formula for the expected number of internal equilibria for a multi-player multi-strategy games for the case of normal payoff entries. We aim to extend the present work to the general case in future publications. In particular, Decartes’ rule of signs for multi-variate polynomials (Itenberg and Roy 1996) might be used to obtain universal estimates, regardless of the underlying payoff distribution.

## Appendix

In this appendix, we present proofs of technical results in previous sections.

### Proof of Lemma 1

The probability distribution, $f_Z$, of $Z=X-Y$ can be found via the joint probability distribution $f_{X,Y}$ as

$$\begin{aligned} f_{Z}(z)=\int _{-\infty }^{\infty } f_{X,Y}(x,x-z)\,dx=\int _{-\infty }^{\infty } f_{X,Y}(y+z,y)\,dy. \end{aligned}$$

Therefore, using the symmetry of $f_{X,Y}$ we get

$$\begin{aligned} f_Z(-z)=\int _{-\infty }^{\infty } f_{X,Y}(x,x+z)\,dx=\int _{-\infty }^{\infty } f_{X,Y}(x+z,x)\,dx=f_Z(z). \end{aligned}$$

If *X* and *Y* are i.i.d with the common probability distribution *f* then

$$\begin{aligned} f_{X,Y}(x,y)=f(x)f(y), \end{aligned}$$

which is symmetric with respect to *x* and *y*, i.e., *X* and *Y* are exchangeable.

### Proof of Proposition 1

We take the sequence of coefficients $(a_0,\ldots , a_{n})$ and move from the left starting from $a_0$ to the right ending at $a_{n}$. When there is a change of sign, we write a 1 and write a 0 when there is not. Then the changes of signs form a binary sequence of length *n*. There are $2^{n}$ of them in total. Thereby $p_{k,n}$ is the probability that there are exactly *k* number 1s in the binary sequence. There are $$\begin{pmatrix} n\\ k \end{pmatrix}$$ such sequences. Since $\{\beta _k\}$ are independent and symmetrically distributed, each sequence has a probability $\frac{1}{2^{n}}$ of occurring. From this we deduce (23).

### Proof of Lemma 2

Since $$\sum \nolimits _{j=0}^{n}\begin{pmatrix} n\\ j \end{pmatrix}(-1)^{j} =(1+(-1))^{n}=0$$, we have

$$\begin{aligned} \sum _{j=k}^{n}\begin{pmatrix} n\\ j \end{pmatrix}(-1)^{j} =-\sum _{j=0}^{k-1}\begin{pmatrix} n\\ j \end{pmatrix}(-1)^{j}. \end{aligned}$$

According to Duong and Tran (2018, Lemma 5.4)

$$\begin{aligned} \sum _{j=0}^{k-1}\begin{pmatrix} n\\ j \end{pmatrix} (-1)^{j}=(-1)^{k-1}\begin{pmatrix} n-1\\ k-1 \end{pmatrix}. \end{aligned}$$

Therefore,

$$\begin{aligned} \sum _{j=k}^{n}\begin{pmatrix} n\\ j \end{pmatrix}(-1)^{j}=(-1)^{k}\begin{pmatrix} n-1\\ k-1 \end{pmatrix}, \end{aligned}$$

or equivalently:

$$\begin{aligned} \sum _{\begin{array}{c} j=k\\ j:~\text {even} \end{array}}^{n}\begin{pmatrix} n\\ j \end{pmatrix}-\sum _{\begin{array}{c} j=k\\ j:~ \text {odd} \end{array}}^{n}\begin{pmatrix} n\\ j \end{pmatrix} =(-1)^{k}\begin{pmatrix} n-1\\ k-1 \end{pmatrix}. \end{aligned}$$

Define $${\bar{S}}_{k,n}:=\sum \nolimits _{j=k}^{n}\begin{pmatrix} n\\ j \end{pmatrix}$$ and $$S_{k,n}:=\sum \nolimits _{j=0}^{k}\begin{pmatrix} n\\ j \end{pmatrix}$$. Then using the property that $$\begin{pmatrix} n\\ j \end{pmatrix}=\begin{pmatrix} n\\ n-j \end{pmatrix}$$ we get ${\bar{S}}_{k,n}=S_{n-k,n}$ and

$$\begin{aligned}&\sum _{\begin{array}{c} j=k\\ j:\text {even} \end{array}}^{n}\begin{pmatrix} n\\ j \end{pmatrix}=\frac{1}{2}\left[ {\bar{S}}_{k,n}+(-1)^{k}\begin{pmatrix} n-1\\ k-1 \end{pmatrix}\right] =\frac{1}{2}\left[ S_{n-k,n}+(-1)^{k}\begin{pmatrix} n-1\\ k-1 \end{pmatrix}\right] ,\\&\sum _{\begin{array}{c} j=k\\ j: \text {odd} \end{array}}^{n}\begin{pmatrix} n\\ j \end{pmatrix}=\frac{1}{2}\left[ {\bar{S}}_{k,n}-(-1)^{k}\begin{pmatrix} n-1\\ k-1 \end{pmatrix}\right] =\frac{1}{2}\left[ S_{n-k,n}-(-1)^{k}\begin{pmatrix} n-1\\ k-1 \end{pmatrix}\right] . \end{aligned}$$

This finishes the proof of this lemma.

### Proof of Proposition 2

The four extreme cases $k\in \{0,1,n-1,n\}$ are special because we can characterise explicitly the events that the sequence $\{a_0,\ldots , a_n\}$ has *k* changes of signs. We have

$$\begin{aligned}&p_{0,n}=\mathbf {P}\Big \{a_0>0,\ldots ,a_n>0\} + \mathbf {P}\{a_0<0,\ldots , a_n<0)\Big \}\\&\qquad =\alpha ^{n+1}+(1-\alpha )^{n+1}.\\&p_{1,n}=\mathbf {P}\Big \{\cup _{k=0}^{n-1}\{a_0>0,\ldots a_k>0, a_{k+1}<0,\ldots , a_n<0\}\\&\qquad \qquad \cup \{a_0<0,\ldots a_k<0, a_{k+1}>0,\ldots , a_n>0\}\Big \}\\&\qquad =\sum _{k=0}^{n-1}\left( \alpha ^{k+1}(1-\alpha )^{n-k}+(1-\alpha )^{k+1}\alpha ^{n-k}\right) \\&\qquad =\alpha (1-\alpha )^n\sum _{k=0}^{n-1}\left( \frac{\alpha }{1-\alpha }\right) ^k+\alpha ^n(1-\alpha )\sum _{k=0}^{n-1}\left( \frac{1-\alpha }{\alpha }\right) ^k\\&\qquad ={\left\{ \begin{array}{ll} \frac{n}{2^n}&{}\text {if}~\alpha =\frac{1}{2},\\ \alpha (1-\alpha )^n\frac{1-\Big (\frac{\alpha }{1-\alpha }\Big )^n}{1-\frac{\alpha }{1-\alpha }}+\alpha ^n(1-\alpha )\frac{1-\Big (\frac{1-\alpha }{\alpha }\Big )^n}{1- \frac{1-\alpha }{\alpha }}&{}\text {if}~\alpha \ne \frac{1}{2} \end{array}\right. }\\&\qquad ={\left\{ \begin{array}{ll} \frac{n}{2^n}&{}\text {if}~\alpha =\frac{1}{2},\\ 2\alpha (1-\alpha )\frac{(1-\alpha )^n-\alpha ^n}{1-2\alpha }&{} \text {if}~\alpha \ne \frac{1}{2}. \end{array}\right. }\\&p_{n,n}=\mathbf {P}\Big \{\{a_0>0,a_1<0,\ldots , (-1)^n a_n>0\}\cup \{a_0<0,a_1>0,\ldots , (-1)^n a_n<0\} \Big \}\\&\qquad ={\left\{ \begin{array}{ll} \alpha ^{\frac{n+2}{2}}(1-\alpha )^{\frac{n}{2}}+(1-\alpha )^{\frac{n+2}{2}}\alpha ^{ \frac{n}{2}}&{}\text {if } n \text { is even},\\ 2 \alpha ^\frac{n+1}{2}(1-\alpha )^\frac{n+1}{2}&{}\text {if } n \text { is odd} \end{array}\right. }\\&\qquad ={\left\{ \begin{array}{ll} \alpha ^{\frac{n}{2}}(1-\alpha )^{\frac{n}{2}}&{}\text {if } n \text { is even},\\ 2 \alpha ^\frac{n+1}{2}(1-\alpha )^\frac{n+1}{2}&{}\text {if } n \text { is odd}. \end{array}\right. } \end{aligned}$$

It remains to compute $p_{n-1,n}$.

$$\begin{aligned} p_{n-1,n}&=\sum _{k=0}^{n-1}\mathbf {P}\Big \{a_k~\text {and}~a_{k+1}~\text {have the same signs}~\text {and there are } n-1 \text { changes of signs in}\\&\quad ~ (a_0,\ldots ,a_k,a_{k+1},\ldots , a_n)\Big \}\\&=:\sum _{k=0}^{n-1}\gamma _{k}. \end{aligned}$$

We now compute $\gamma _k$. This depends on the parity of *n* and *k*. If both *n* and *k* are even, then

$$\begin{aligned} \gamma _k&=\mathbf {P}\Big (a_0>0,a_1<0,\ldots , a_k>0, a_{k+1}>0,\ldots a_n<0\Big )\\&\quad +\mathbf {P}\Big (a_0<0,a_1>0,\ldots , a_k<0, a_{k+1}<0,\ldots a_n>0\Big )\\&=(1-\alpha )^\frac{n}{2}\alpha ^\frac{n+2}{2}+(1-\alpha )^{\frac{n+2}{2}}\alpha ^\frac{n}{2}. \end{aligned}$$

If *n* is even and *k* is odd, then

$$\begin{aligned} \gamma _k&=\mathbf {P}\Big (a_0>0,a_1<0,\ldots , a_k<0, a_{k+1}<0,\ldots a_n<0\Big )\\&\quad +\mathbf {P}\Big (a_0<0,a_1>0,\ldots , a_k>0, a_{k+1}>0,\ldots a_n>0\Big )\\&=\alpha ^\frac{n+2}{2}(1-\alpha )^\frac{n}{2}+(1-\alpha )^\frac{n+2}{2}\alpha ^\frac{n}{2}. \end{aligned}$$

Therefore, in both cases, i.e., if *n* is even we get

$$\begin{aligned} \gamma _k=\alpha ^\frac{n}{2}(1-\alpha )^\frac{n}{2}. \end{aligned}$$

From this we deduce $p_{n-1,n}= n \alpha ^\frac{n}{2}(1-\alpha )^\frac{n}{2}$. Similarly if *n* is odd and *k* is even

$$\begin{aligned} \gamma _k&=\mathbf {P}\Big (a_0>0,a_1<0,\ldots , a_k>0, a_{k+1}>0,\ldots a_n>0\Big )\\&\quad +\mathbf {P}\Big (a_0<0,a_1>0,\ldots , a_k<0, a_{k+1}<0,\ldots a_n<0\Big )\\&=(1-\alpha )^{\frac{n+3}{2}}\alpha ^\frac{n-1}{2}+(1-\alpha )^\frac{n-1}{2}\alpha ^\frac{n+3}{2}. \end{aligned}$$

If both *n* and *k* are odd

$$\begin{aligned} \gamma _k&=\mathbf {P}\Big (a_0>0,a_1<0,\ldots , a_k<0, a_{k+1}<0,\ldots a_n>0\Big )\\&\quad +\mathbf {P}\Big (a_0<0,a_1>0,\ldots , a_k>0, a_{k+1}>0,\ldots a_n<0\Big )\\&=\alpha ^\frac{n+1}{2}(1-\alpha )^\frac{n+1}{2}+(1-\alpha )^\frac{n+1}{2}\alpha ^\frac{n+1}{2}. \end{aligned}$$

Then when *n* is odd, we obtain

$$\begin{aligned} p_{n-1,n}&=\frac{n+1}{2}\Big [(1-\alpha )^{\frac{n+3}{2}}\alpha ^\frac{n-1}{2}+(1-\alpha )^\frac{n-1}{2}\alpha ^\frac{n+3}{2}\Big ]+(n-1)\alpha ^\frac{n+1}{2}(1-\alpha )^\frac{n+1}{2}\\&=\alpha ^\frac{n+1}{2}(1-\alpha )^\frac{n+1}{2}\left[ \frac{n+1}{2}\left( \frac{\alpha }{1-\alpha }+\frac{1-\alpha }{\alpha }\right) +(n-1)\right] . \end{aligned}$$

In conclusion,

$$\begin{aligned} p_{n-1,n}={\left\{ \begin{array}{ll} n \alpha ^\frac{n}{2}(1-\alpha )^\frac{n}{2}&{}\text {if } n \text { even},\\ \alpha ^\frac{n+1}{2}(1-\alpha )^\frac{n+1}{2}\bigg [\frac{n+1}{2}\Big ( \frac{\alpha }{1-\alpha }+\frac{1-\alpha }{\alpha }\Big )+(n-1)\bigg ]&{}\text {if } n \text { odd}. \end{array}\right. } \end{aligned}$$

### Proof of Lemma 4

Applying the law of total probability

$$\begin{aligned} \mathbf {P}(A|B)=\mathbf {P}(A|B,C)\mathbf {P}(C|B)+\mathbf {P}(A|B,\bar{C})\mathbf {P}(\bar{C}|B), \end{aligned}$$

we have:

$$\begin{aligned}&\mathbf {P}\Big (k~\text {sign switches in } \{a_0,\ldots ,a_n\}\big \vert a_{n}>0\Big )\\&\quad =\mathbf {P}\Big (k~\text { sign switches in}~\{a_0,\ldots , a_n\}\big \vert a_{n}>0, a_{n-1}>0)\mathbf {P}(a_{n-1}>0|a_{n}>0\Big )\\&\qquad +\mathbf {P}\Big (k~\text {sign switches in }\{a_0,\ldots ,a_n\}\big \vert a_{n}>0,a_{n-1}<0)\mathbf {P}(a_{n-1}<0|a_{n}>0\Big ). \end{aligned}$$

Since $a_{n-1}$ and $a_{n}$ are independent, we have $\mathbf {P}(a_{n-1}>0\big \vert a_{n}>0)=\mathbf {P}(a_{n-1}>0)$ and $\mathbf {P}(a_{n-1}<0\big \vert a_{n}>0)=\mathbf {P}(a_{n-1}<0)$. Therefore,

$$\begin{aligned}&\mathbf {P}\Big (k~\text { sign switches in}~\{a_0,\ldots ,a_n\}\big \vert a_{n}>0\Big )\\&\qquad = \mathbf {P}\Big (k~\text {sign switches in}~\{a_0,\ldots ,a_n\}\big \vert a_{n}>0,a_{n-1}>0\Big )\mathbf {P}(a_{n-1}>0)\\&\qquad \quad +\mathbf {P}\Big (k\text { sign switches in}~\{a_0,\ldots ,a_n\}\big \vert a_{n}>0,a_{n-1}<0\Big )\mathbf {P}(a_{n-1}<0)\\&\qquad =\mathbf {P}\Big (k~\text { sign switches in}~\{a_0,\ldots ,a_{n-1}\}\big \vert a_{n-1}>0\Big )\mathbf {P}(a_{n-1}>0)\\&\qquad \quad +\mathbf {P}\Big (k-1~\text {sign switches in}~\{a_0,\ldots ,a_{n-1}\}\big \vert a_{n-1}<0\Big )\mathbf {P}(a_{n-1}<0). \end{aligned}$$

Therefore we obtain the first relationship in (33). The second one is proved similarly.

### Proof of Lemma 5

From (33), it follows that

35 $$\begin{aligned} v_{k-1,n-1}=\frac{u_{k,n}-\alpha u_{k,n-1}}{1-\alpha },\quad v_{k,n-1}=\frac{u_{k+1,n}-\alpha u_{k+1,n-1}}{1-\alpha }. \end{aligned}$$

Substituting (35) into (33) we obtain

$$\begin{aligned} \frac{u_{k+1,n+1}-\alpha u_{k+1,n}}{1-\alpha }=\alpha u_{k-1,n-1}+(1-\alpha )\frac{u_{k+1,n}-\alpha u_{k+1,n-1}}{1-\alpha }, \end{aligned}$$

which implies that

$$\begin{aligned} u_{k+1,n+1}&=(1-\alpha )\alpha u_{k-1,n-1}+(1-\alpha )(u_{k+1,n}-\alpha u_{k+1,n-1})+\alpha u_{k+1,n} \\&=(1-\alpha )\alpha u_{k-1,n-1}-\alpha (1-\alpha )u_{k+1,n-1}+u_{k+1,n}. \end{aligned}$$

Re-indexing we get $u_{k,n} =(1-\alpha )\alpha (u_{k-2,n-2}-u_{k,n-2})+u_{k,n-1}$. Similarly we obtain the recursive formula for $v_{k,n}$.

### Proof of Proposition 3

From Lemmas 4 and 5 we have

$$\begin{aligned} p_{k,n}&=\alpha u_{k,n}+(1-\alpha )v_{k,n}\\&= \alpha [\alpha (1-\alpha )(u_{k-2,n-2}-u_{k,n-2})+u_{k,n-1}]\\&\quad +(1-\alpha )[\alpha (1-\alpha )(v_{k-2,n-2}-v_{k,n-2})+v_{k,n-1}]\\&= \alpha (1-\alpha )[\alpha (u_{k-2,n-2}-u_{k,n-2})+(1-\alpha )(v_{k-2,n-2}-v_{k,n-2})]\\&\quad +\alpha u_{k,n-1}+(1-\alpha )v_{k,n-1}\\&= \alpha (1-\alpha )(p_{k-2,n-2}-p_{k,n-2})+p_{k,n-1}. \end{aligned}$$

This finishes the proof.

### Proof of Theorem 7

Set $1/A^2:=\alpha (1-\alpha )$. By the Cauchy–Schwarz inequality $\alpha (1-\alpha )\le \frac{(\alpha +1-\alpha )^2}{4}=\frac{1}{4}$, it follows that $A^2\ge 4$. Define $a_{k,n}:=A^n p_{k,n}$. Substituting this relation into (34) we get the following recursive formula for $a_{k,n}$

$$\begin{aligned} a_{k,n}=a_{k-2,n-2}-a_{k,n-2}+A a_{k,n-1}. \end{aligned}$$

According to Proposition 2

36 $$\begin{aligned} a_{0,n}&=A^n p_{0,n}=A^n\Big (\alpha ^{n+1}+(1-\alpha )^{n+1}\Big )=\alpha \left( \frac{\alpha }{1-\alpha }\right) ^\frac{n}{2}+(1-\alpha )\left( \frac{1- \alpha }{\alpha }\right) ^\frac{n}{2}, \end{aligned}$$

37 $$\begin{aligned} a_{1,n}&=A^n p_{1,n}={\left\{ \begin{array}{ll} n&{}\text {if}~~\alpha =\frac{1}{2},\\ \frac{2\alpha (1-\alpha )}{1-2\alpha }\Big [\big (\frac{1-\alpha }{ \alpha }\big )^\frac{n}{2}-\big (\frac{\alpha }{1-\alpha }\big )^\frac{n}{2}\Big ].&{} \end{array}\right. } \end{aligned}$$

Also $a_{k,n}=0$ for $k>n$. Let *F*(*x*, *y*) be the generating function of $a_{k,n}$, that is

$$\begin{aligned} F(x,y):=\sum _{k=0}^\infty \sum _{n=0}^\infty a_{k,n}x^k y^n. \end{aligned}$$

Define

$$\begin{aligned} g(x,y)=\sum _{n=0}^\infty a_{0,n} y^n+\sum _{n=0}^\infty a_{1,n} x y^n. \end{aligned}$$

From (36) and (37) we have: for $\alpha =\frac{1}{2}$

$$\begin{aligned} g(x,y)=\sum _{n=0}^\infty y^n+xy\sum _{n=0}^\infty ny^{n-1}=\frac{1}{1-y}+xy\frac{d}{dy}\left( \frac{1}{1-y}\right) =\frac{1-y+xy}{(1-y)^2}, \end{aligned}$$

and for $$\alpha \ne \frac{1}{2}$$

$$\begin{aligned}&g(x,y)\\&\quad =\sum _{n=0}^\infty \left[ \alpha \left( \frac{\alpha }{1-\alpha }\right) ^\frac{n}{2}+(1-\alpha )\left( \frac{1-\alpha }{\alpha }\right) ^\frac{n}{2}\right] y^n+\frac{2\alpha (1-\alpha )x}{1-2\alpha } \sum _{n=1}^\infty \left[ \left( \frac{1-\alpha }{\alpha }\right) ^\frac{n}{2}-\left( \frac{\alpha }{1-\alpha }\right) ^\frac{n}{2}\right] \, y^n\\&\quad =\left[ \alpha -\frac{2\alpha (1-\alpha )x}{1-2\alpha }\right] \sum _{n=0}^\infty \left( \frac{\alpha }{1-\alpha }\right) ^\frac{n}{2}\,y^n+\left[ 1-\alpha +\frac{2\alpha (1-\alpha )x}{1-2\alpha }\right] \sum _{n=0}^\infty \left( \frac{1-\alpha }{\alpha }\right) ^\frac{n}{2}\, y^n\\&\quad =\left[ \alpha -\frac{2\alpha (1-\alpha )x}{1-2\alpha }\right] \sum _{n=0}^\infty (\alpha A)^n y^n+\left[ 1-\alpha +\frac{2\alpha (1-\alpha )x}{1-2\alpha }\right] \sum _{n=0}^\infty ((1-\alpha )A)^n y^n\\&\quad =\left[ \alpha -\frac{2\alpha (1-\alpha )x}{1-2\alpha }\right] \frac{1}{1-\alpha A y}+\left[ 1-\alpha +\frac{2\alpha (1-\alpha )x}{1-2\alpha }\right] \frac{1}{1-(1-\alpha )A y}\\&\quad =\frac{\left( \alpha (1-2\alpha )-2\alpha (1-\alpha )x\right) \left( 1-(1-\alpha )Ay\right) +\left( (1-\alpha )(1-2\alpha )+2\alpha (1-\alpha )x\right) \left( 1-\alpha Ay\right) }{(1-2\alpha )(1-\alpha y)(1-(1-\alpha )Ay)}\\&\quad =\frac{1-\frac{2y}{A}+\frac{2xy}{A}}{1-Ay+y^2}. \end{aligned}$$

Note that in the above computations we have the following identities

$$\begin{aligned} \frac{1}{A^2}\!=\!\alpha (1-\alpha ),\quad \frac{\alpha }{1-\alpha }\!=\!(\alpha A)^2,\quad \frac{1-\alpha }{\alpha }\!=\!(1-\alpha )^2A^2,\quad (1-\alpha Ay)(1-(1-\alpha )Ay)\!=\!1-Ay+y^2. \end{aligned}$$

Now we have

38 $$\begin{aligned} F(x,y)&=\sum _{k=0}^\infty \sum _{n=0}^\infty a_{k,n}x^k y^n\nonumber \\&=g(x,y)+\sum _{k=2}^\infty \sum _{n=2}^\infty (a_{k-2,n-2}-a_{k,n-2}+A a_{k,n-1})x^k y^n\nonumber \\&=g(x,y)+\sum _{k=2}^\infty \sum _{n=2}^\infty a_{k-2,n-2} x^k y^n-\sum _{k=2}^\infty \sum _{n=2}^\infty a_{k,n-2} x^k y^n+ A \sum _{k=2}^\infty \sum _{n=2}^\infty a_{k,n-1}x^k y^n \end{aligned}$$

39 $$\begin{aligned}&=g(x,y)+(I)+(II)+(III). \end{aligned}$$

We rewrite the sums (I), (II) and (III) as follow. For the first sum

$$\begin{aligned} (I)=\sum _{k=2}^\infty \sum _{n=2}^\infty a_{k-2,n-2} x^k y^n=x^2y^2\sum _{k=0}^\infty \sum _{n=0}^\infty a_{k,n} x^k y^n=x^2y^2 F(x,y). \end{aligned}$$

For the second sum

$$\begin{aligned} (II)=\sum _{k=2}^\infty \sum _{n=2}^\infty a_{k,n-2} x^k y^n&=\sum _{k=0}^\infty \sum _{n=2}^\infty a_{k,n-2} x^k y^n-\sum _{n=2}^\infty a_{0,n-2}y^n-\sum _{n=2}^\infty a_{1,n-2} x y^n\\&=y^2 \sum _{k=0}^\infty \sum _{n=0}^\infty a_{k,n} x^k y^n-y^2\sum _{n=0}^\infty a_{0,n}y^n-y^2\sum _{n=1}^\infty a_{1,n} xy^n\\&=y^2 (F(x,y)-g(x,y)). \end{aligned}$$

And finally for the last sum

$$\begin{aligned} (III)=\sum _{k=2}^\infty \sum _{n=2}^\infty a_{k,n-1} x^k y^n&=y(F(x,y)-g(x,y)). \end{aligned}$$

Substituting these sums back into (39) we get

$$\begin{aligned} F(x,y)\!=\!g(x,y)+x^2y^2 F(x,y)\!-\!y^2 (F(x,y)-g(x,y))\!+\!A y(F(x,y)-g(x,y)), \end{aligned}$$

which implies that

$$\begin{aligned} F(x,y)=\frac{g(x,y)(1-Ay+y^2)}{(1-Ay+y^2-x^2y^2)}. \end{aligned}$$

For $\alpha =\frac{1}{2}$, we get

$$\begin{aligned} F(x,y)&=\frac{1-y+xy}{(1-y)^2}\frac{(1-y)^2}{(1-y)^2-x^2y^2}=\frac{1}{1-y-xy}\\&=\sum _{n=0}^\infty (1+x)^n y^n\\&=\sum _{n=0}^\infty \sum _{k=0}^n \begin{pmatrix} n\\ k \end{pmatrix} x^k y^n, \end{aligned}$$

which implies that $$\alpha _{k,n}=\begin{pmatrix} n\\ k \end{pmatrix}$$. Hence for the case $\alpha =\frac{1}{2}$, we obtain $$p_{k,n}=\frac{1}{2^n}\begin{pmatrix} n\\ k \end{pmatrix}$$.

For the case $\alpha \ne \frac{1}{2}$ we obtain

$$\begin{aligned} F(x,y)=\frac{1-\frac{2y}{A}+\frac{2xy}{A}}{1-Ay+y^2}\frac{1-Ay+y^2}{1-Ay+y^2-x^2y^2}=\frac{1-\frac{2y}{A}+\frac{2xy}{A}}{1-Ay+y^2-x^2y^2}. \end{aligned}$$

Finding the series expansion for this case is much more involved than the previous one. Using the multinomial theorem we have

$$\begin{aligned} \frac{1}{1-Ay+y^2-x^2y^2}&=\sum _{m=0}^\infty (x^2y^2-y^2+Ay)^m\\&=\sum _{m=0}^\infty ~\sum _{\begin{array}{c} 0\le i,j,l\le m\\ i+j+l=m \end{array}}\begin{pmatrix} m\\ i,j,l \end{pmatrix}(x^2y^2)^i(-y^2)^j(Ay)^l \\&=\sum _{m=0}^\infty ~\sum _{\begin{array}{c} 0\le i,j,l\le m\\ i+j+l=m \end{array}}\begin{pmatrix} m\\ i,j,l \end{pmatrix}(-1)^jA^l x^{2i}y^{2i+2j+l} \\&=\sum _{m=0}^\infty ~\sum _{\begin{array}{c} 0\le i,l\le m\\ i+l\le m \end{array}}\begin{pmatrix} m\\ i,m-i-l,l \end{pmatrix}(-1)^{m-i-l}A^l x^{2i}y^{2m-l}. \end{aligned}$$

Therefore

40 $$\begin{aligned} F(x,y)&=\frac{1}{A}(A-2y+2xy)\sum _{m=0}^\infty ~\sum _{\begin{array}{c} 0\le i,l\le m\\ i+l\le m \end{array}}\begin{pmatrix} m\\ i,m-i-l,l \end{pmatrix}(-1)^{m-i-l}A^l x^{2i}y^{2m-l}\nonumber \\&=\sum _{m=0}^\infty ~\sum _{\begin{array}{c} 0\le i,l\le m\\ i+l\le m \end{array}}\begin{pmatrix} m\\ i,m-i-l,l \end{pmatrix}(-1)^{m-i-l}A^{l-1} \Big ( A x^{2i}y^{2m-l}-2x^{2i}y^{2m-l+1}\nonumber \\&\quad +2x^{2i+1}y^{2m-l+1}\Big ). \end{aligned}$$

From this we deduce that:

If *k* is even, $k=2k'$, then to obtain the coefficient of $x^ky^n$ on the right-hand side of (40), we select (*i*, *m*, *l*) such that

$$ \begin{aligned} (i=k'~ \& ~ 2m-l=n ~ \& ~0\le i, l\le m)\quad \text {or}\quad (i=k'~ \& ~ 2m-l+1=n~ \& ~0\le i, l\le m). \end{aligned}$$

Then we obtain

$$\begin{aligned} a_{k,n}&=\sum _{m=\lceil \frac{n}{2}\rceil }^n\begin{pmatrix} m\\ k',m-k'-(2m-n),2m-n \end{pmatrix}(-1)^{m-k'-(2m-n)}A^{2m-n} \\&\quad + 2\,\sum _{m=\lceil \frac{n-1}{2}\rceil }^n\begin{pmatrix} m\\ k',m-k'-(2m-n+1),2m-n+1 \end{pmatrix}(-1)^{m-k'-(2m-n+1)+1} A^{2m-n} \\&=\sum _{m=\lceil \frac{n}{2}\rceil }^n\begin{pmatrix} m\\ k',n-k'-m,2m-n \end{pmatrix}(-1)^{n-k'-m}A^{2m-n} \\&\qquad + 2\,\sum _{m=\lceil \frac{n-1}{2}\rceil }^n\begin{pmatrix} m\\ k', n-k'-m-1,2m-n+1 \end{pmatrix}(-1)^{n-k'-m} A^{2m-n} \\&={\left\{ \begin{array}{ll} \sum \nolimits _{m=\lceil \frac{n}{2}\rceil }^n\left[ \begin{pmatrix} m\\ k',n-k'-m,2m-n \end{pmatrix}+2\begin{pmatrix} m\\ k', n-k'-m-1,2m-n+1 \end{pmatrix}\right] \\ \quad \times (-1)^{n-k'-m}A^{2m-n}&{}\text {if } n \text { even},\\ \sum \nolimits _{m=\lceil \frac{n}{2}\rceil }^n\left[ \begin{pmatrix} m\\ k',n-k'-m,2m-n \end{pmatrix}+2\begin{pmatrix} m\\ k', n-k'-m-1,2m-n+1 \end{pmatrix}\right] \\ \qquad \times (-1)^{n-k'-m}A^{2m-n}+2\begin{pmatrix} \lceil \frac{n-1}{2}\rceil \\ k' \end{pmatrix}(-1)^{\lceil \frac{n-1}{2}\rceil -k'+1} A^{-1}&\text {if } n \text { odd} \end{array}\right. } \\&={\left\{ \begin{array}{ll} \sum \nolimits _{m=\lceil \frac{n}{2}\rceil }^n \frac{n-k+1}{2m-n+1}\begin{pmatrix} m\\ k',n-k'-m,2m-n \end{pmatrix}(-1)^{n-k'-m}A^{2m-n}&{}\text {if } n \text { even},\\ \sum \nolimits _{m=\lceil \frac{n}{2}\rceil }^n \frac{n-k+1}{2m-n+1}\begin{pmatrix} m\\ k',n-k'-m,2m-n \end{pmatrix}(-1)^{n-k'-m}A^{2m-n}\\ \quad +\,2\begin{pmatrix} \lceil \frac{n-1}{2}\rceil \\ k' \end{pmatrix}(-1)^{\lceil \frac{n-1}{2}\rceil -k'+1} A^{-1}&\text {if } n \text { odd}. \end{array}\right. } \end{aligned}$$

Similarly, if *k* is odd, $k=2k'+1$, then to obtain the coefficient of $x^ky^n$ on the right-hand side of (40), we select (*i*, *m*, *l*) such that

$$ \begin{aligned} (i=k'~ \& ~ 2m-l+1=n~ \& ~0\le i, l\le m), \end{aligned}$$

and obtain

$$\begin{aligned} a_{k,n}=2\,\sum _{m=\lceil \frac{n-1}{2}\rceil }^n\begin{pmatrix} m\\ k', n-k'-m-1,2m-n+1 \end{pmatrix}(-1)^{n-k'-m-1} A^{2m-n}. \end{aligned}$$

From $a_{k,n}$ we compute $p_{k,n}$ using the relations $p_{k,n}=\frac{a_{k,n}}{A^n}$ and $A^2=\frac{1}{\alpha (1-\alpha )}$ and obtain the claimed formulas. This finishes the proof of this theorem.

#### Remark 4

We can find $a_{k,n}$ by establishing a recursive relation. We have

$$\begin{aligned} \frac{1}{F(x,y)}&=\frac{1-Ay+y^2-x^2y^2}{1-\frac{2y}{A}+\frac{2xy}{A}}\\&=-\frac{Axy}{2}-\frac{Ay}{2}+\frac{A^2}{4}+\frac{1-A^2/4}{1-\frac{2y}{A}+\frac{2xy}{A}}\\&=-\frac{Axy}{2}-\frac{Ay}{2}+\frac{A^2}{4}+(1-A^2/4)\sum _{n=0}^\infty \left( \frac{2y}{A}(1-x)\right) ^n\\&=-\frac{Axy}{2}-\frac{Ay}{2}+\frac{A^2}{4}+(1-A^2/4)\sum _{n=0}^\infty \left( \frac{2}{A}\right) ^n (1-x)^ny^n\\&=-\frac{Axy}{2}-\frac{Ay}{2}+\frac{A^2}{4}+(1-A^2/4)\sum _{n=0}^\infty \sum _{k=0}^n (-1)^k C_{k,n}\left( \frac{2}{A}\right) ^n x^ky^n\\&=1+\left( \frac{2}{A}-A\right) y-\frac{2}{A}xy+(1-A^2/4)\sum _{n=2}^\infty \sum _{k=0}^n (-1)^k C_{k,n}\left( \frac{2}{A}\right) ^n x^ky^n\\&=:\sum _{n=0}^\infty \sum _{k=0}^n b_{k,n}x^k y^n:=B(x,y). \end{aligned}$$

where

$$\begin{aligned}&b_{0,0}=1, \quad b_{0,1}=\frac{2}{A}-A, \quad b_{1,1}=-\frac{2}{A}\quad \text {and} \\&b_{k,n}=(1-A^2/4)(-1)^k C_{k,n}\Big (\frac{2}{A}\Big )^n \quad \text {for}~~0\le k \le n, n\ge 2. \end{aligned}$$

Using the relation that

$$\begin{aligned} F(x,y)B(x,y)=\left( \sum _{n=0}^\infty \sum _{k=0}^\infty a_{k,n} x^k y^n\right) \left( \sum _{n'=0}^\infty \sum _{k'=0}^\infty b_{k'n'} x^{k'} y^{n'}\right) =1, \end{aligned}$$

we get the following recursive formula to determine $a_{K,N}$

$$\begin{aligned} a_{0,0}=\frac{1}{b_{0,0}}=1, \quad a_{0,N}=-\sum _{n=0}^{N-1}a_{0,n}b_{0,N-n} , \quad a_{K,N}=-\sum _{k=0}^{K-1}\sum _{n=0}^{N-1} a_{k,n}b_{K-k,N-n}. \end{aligned}$$

It is not trivial to obtain an explicit formula from this recursive formula. However, it is easily implemented using a computational software such as Mathematica or Mathlab.
